# The Lipidomic Profile Discriminates Between MASLD and MetALD

**DOI:** 10.1111/apt.70012

**Published:** 2025-02-11

**Authors:** Kai Markus Schneider, Feng Cao, Helen Ye Rim Huang, Lanlan Chen, Yazhou Chen, Rongpeng Gong, Anastasia Raptis, Kate Townsend Creasy, Jan Clusmann, Felix van Haag, Paul‐Henry Koop, Adrien Guillot, Tom Luedde, Rohit Loomba, Sven Francque, Carolin Victoria Schneider

**Affiliations:** ^1^ Department of Internal Medicine III, Gastroenterology, Metabolic Diseases and Intensive Care University Hospital RWTH Aachen Aachen Germany; ^2^ Department of Medicine I, Deptartment of Gastroenterology and Hepatology, Faculty of Medicine and University Hospital Carl Gustav Carus TUD Dresden University of Technology Dresden Germany; ^3^ Center for Regenerative Therapies Dresden (CRTD) Technische Universität (TU) Dresden Dresden Germany; ^4^ Else Kroener Fresenius Center for Digital Health, Faculty of Medicine and University Hospital Carl Gustav Carus TUD Dresden University of Technology Dresden Germany; ^5^ Department of Hepatology & Gastroenterology, Campus Virchow‐Klinikum and Campus Charité Mitte Charité – Universitätsmedizin Berlin Berlin Germany; ^6^ Department of Biobehavioral Health Sciences, School of Nursing University of Pennsylvania Philadelphia Pennsylvania USA; ^7^ Department of Gastroenterology, Hepatology and Infectious Diseases, Medical Faculty Heinrich‐Heine‐University Düsseldorf Germany; ^8^ MASLD Research Center, Division of Gastroenterology and Hepatology, Department of Medicine University of California at San Diego San Diego California USA; ^9^ Department of Gastroenterology Hepatology Antwerp University Hospital Edegem Belgium; ^10^ InflaMed Centre of Excellence, Laboratory for Experimental Medicine and Paediatrics, Translational Sciences in Inflammation and Immunology, Faculty of Medicine and Health Sciences University of Antwerp Wilrijk Belgium; ^11^ The Institute for Translational Medicine and Therapeutics, the Perelman School of Medicine University of Pennsylvania Philadelphia Pennsylvania USA

**Keywords:** ethanol metabolism, HDL, Lipidomics, liver disease, MASLD, MetALD, proton density fat fraction, UK biobank

## Abstract

**Background:**

The recent consensus statement redefined steatotic liver diseases. Metabolic dysfunction‐associated steatotic liver disease (MASLD) and metabolic dysfunction and alcohol‐related liver disease (MetALD) now represent distinct disease entities. However, biomarkers that differentiate MASLD and MetALD remain largely unknown.

**Aims:**

To identify lipidomic biomarkers with discriminatory potential for distinguishing MetALD from MASLD.

**Methods:**

Using the UK Biobank dataset, 40,534 people with available MRI liver scans were analysed. A total of, 11,217 cases with a proton density fat fraction (PDFF) ≥ 5% were identified as having steatotic liver disease. Among these, lipidomic profiles were obtained for 5539 MASLD and 462 MetALD cases. A total of, 250 plasma lipidomic and metabolomic parameters were analysed. Mendelian randomisation (MR) analysis was used to confirm the association between alcohol consumption and the lipidomic biomarkers.

**Results:**

When comparing the top 30 differentially expressed lipidomic biomarkers predicting MetALD compared to MASLD, the majority were related to HDL and were significantly overrepresented at both analysed time points. The top five metabolites were: acetoacetate, 3‐hydroxybutyrate, phospholipids in Large HDL, concentration of large HDL particles, free cholesterol in large HDL. The sensitivity analysis comparing alcohol‐related liver disease to MASLD revealed similar associations, suggesting that the HDL signature is stable over time. Additionally, MR analysis further confirmed that alcohol consumption was associated with increased levels of HDL‐related metabolites.

**Conclusion:**

Our findings indicate that HDL‐centric lipidomic markers, particularly those within the larger and medium HDL subfraction, may differentiate MetALD from MASLD. Further longitudinal and experimental studies are warranted to validate these findings and assess their clinical implications.

## Graphical abstract

HDL‐centric lipidomic biomarkers, particularly within large and medium HDL subfractions, distinguish metabolic dysfunction and alcohol‐related liver disease from metabolic dysfunction‐associated steatotic liver disease, suggesting potential biomarkers for clinical differentiation.

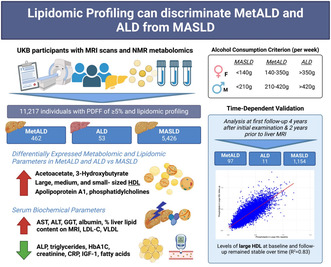

## 1 Introduction

With the novel multi‐society Delphi consensus statement, Metabolic dysfunction‐associated steatotic liver disease (MASLD) and combined metabolic dysfunction‐and alcohol‐related steatotic liver disease (MetALD) are emerging as distinct subsets of steatotic liver disease (SLD) [1]. Both conditions are defined by lipid accumulation in the hepatocytes and by metabolic alterations that are driven by an interplay between genetics, lifestyle and environmental factors [2, 3]. MASLD is considered a consequence of components of the metabolic syndrome, characterised by overweight/obesity, dyslipidemia, insulin resistance and hypertension [4, 5]. Mild consumption of alcohol is accepted within the MASLD diagnosis. By contrast, MetALD includes the additional component of moderate alcohol consumption, making it a unique entity that blurs the lines between metabolic liver disease and alcohol‐related liver damage [6, 7, 8, 9].

One of the challenges in SLD management is the accurate classification and distinction of these novel conditions [10]. Currently, the main factor used to distinguish MASLD from MetALD is self‐reported alcohol consumption [1]. However, self‐reporting is notoriously unreliable due to various reasons, such as recall bias, social desirability bias and the stigmatisation associated with excessive alcohol consumption [11, 12]. Moreover, individuals may be unaware of the actual amount of alcohol they consume, particularly when it comes to the alcoholic content in various mixed beverages. Hence, there is a clinical need for objective, quantifiable markers that can differentiate between these two conditions reliably.

The increasing availability of omics technologies, particularly lipidomics, provides a promising avenue for identifying such markers. Lipidomics involves the large‐scale study of lipids in biological systems and can provide a nuanced understanding of metabolic pathways, signalling networks and disease processes [13]. Previous research has already shown distinct lipidomic profiles in patients with non‐alcoholic fatty liver disease (NAFLD) and alcohol‐related liver disease (ALD), but little is known about the lipidomic distinctions between MASLD and MetALD [14, 15].

The UK Biobank, one of the most comprehensive biomedical databases available, offers a unique opportunity to explore these plasma lipidomic profiles in a large‐scale, population‐based setting. Therefore, this study aims to use the UK Biobank liver MRI dataset to analyse the lipidomic profiles of individuals with MASLD and MetALD, and ALD. By doing so, we seek to identify potential lipidomic biomarkers that could offer more reliable and objective criteria for differentiating these two closely related but distinct liver conditions.

## 2 Methods

### 2.1 Study Design and Dataset

This study is a cohort study utilising data from the UK Biobank, a large, population‐based study. The present study focuses on 40,534 individuals who had undergone MRI scans to assess liver fat at the second follow‐up. All participants were registered with the UK National Health Service and attended an initial examination, which was followed by a long‐term follow‐up that takes place continuously. Demographic and clinical characteristics, including age, gender and body mass index (BMI), were recorded for all participants at the time of liver MRI.

### 2.2 Inclusion and Exclusion Criteria

**FIGURE 1:**
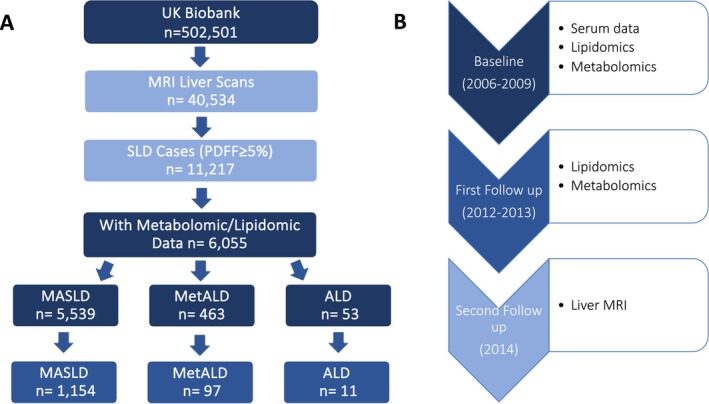
(A) Flow chart of patient inclusion and (B) timeline of the study. ALD, alcohol‐related liver disease, SLD, steatotic liver disease; MASLD, metabolic dysfunction‐associated steatotic liver disease; MetALD, metabolic dysfunction‐associated alcohol‐related liver disease; alcohol‐related liver disease. The Colours of the boxes indicate the different time points (baseline, follow‐up 1 and follow‐up 2).

A proton density fat fraction (PDFF) of ≥ 5% was used as the criterion to identify SLD. The categorisation into MASLD and MetALD was described here [9]. Plasma lipidomic and metabolomic profiles were available for 5539 MASLD, 462 MetALD and 53 ALD cases that had also undergone MRI of the liver (Figure 1A). MASLD was defined as alcohol consumption below 140 g/week for females and 210 g/week for males, and MetALD was defined as alcohol consumption between 140 and 350 g/week for females and 210 and 420 g/week for males. For the sensitivity analysis, the ALD group was defined as alcohol consumption above 350 g/week for females and 420 g/week for males [9].

### 2.3 Lipidomic and Metabolomic Profiling

Nightingale Health used high‐throughput nuclear magnetic resonance (NMR) spectroscopy to quantify 249 metabolic measures per EDTA plasma sample. These measures include 168 absolute levels and 81 ratios, covering cholesterol metabolism, fatty acid compositions and various low‐molecular weight metabolites at two time points. Lipidomic and metabolomic profiling was performed at baseline and for a subset of patients (Figure 1B) at follow‐up. The subset at follow‐up included 1154 MASLD, 97 MetALD and 11 ALD patients. Serum biochemical parameters, including liver enzymes, were measured at baseline. Before analysis, the lipidomic data underwent rigorous quality control to remove outliers and normalise the distributions.

### 2.4 Statistical Analysis

Statistical analyses were conducted to compare the baseline characteristics and serum biochemical parameters between MASLD and MetALD participants. Differences in continuous variables were assessed using the t‐test, while categorical variables were compared using the chi‐square test. Prior to analysis, each metabolite level was divided by its respective mean value to achieve normalisation, resulting in a dimensionless scale that ensured comparability across markers while preserving their relative differences. Differential expression of lipidomic markers between MASLD and MetALD was determined using regression models. Effect sizes were calculated as odds ratios (OR) for each marker. A subset of participants had follow‐up lipidomic analyses performed approximately 4 years after the initial examination and 2 years prior to the liver MRI. The stability of lipidomic markers over time was assessed by correlating levels of acetoacetate and phospholipids in large HDL particles between the two time points. Cubic splines were constructed to explore the relationship between key metabolites (HDL and acetoacetate) and the risk of MetALD. The associations between exposure and outcome were analysed using restricted‐cubic‐spline plots to explore the shape of the association between the metabolites and MetALD, fitting a restricted‐cubic‐spline function with four knots (at the 5th, 35th, 65th and 95th percentiles). The spline curves were truncated at the 5th and 95th percentiles of the distribution curve. Significance was determined at *p* < 0.05 unless otherwise specified. For lipidomic analyses, the Bonferroni method was employed to correct for multiple comparisons (*p* < 0.05/249).

### 2.5 Publicly Available Metabolomics Data Analysis

We performed an overrepresentation enrichment analysis on the top discriminatory metabolites from MetALD in the MetaboAnalyst interface, mapping these to 3694 metabolite and lipid pathways from the Relational Database of Metabolomics Pathway (RAMP‐DB) [16]. The enrichment analysis utilised hypergeometric tests to evaluate whether a particular metabolite set is represented more than expected by chance in the given compound list. The one‐tailed *p*‐values are provided as their raw value, as well as adjustments for false discovery rate and Holms‐Bonferroni method.

### 2.6 Mendelian Randomisation

Due to confounding factors, it is difficult to establish a causal relationship between certain metabolic signatures and disease phenotypes in population‐based cohorts. Mendelian randomisation (MR) analysis is a research method that utilises genetic variations as instrumental variables, enabling effective control of confounding factors and providing more robust causal inference [17, 18, 19]. The causal relationship between alcohol consumption and specific metabolites remains unclear. MR allows us to study the potential impact of alcohol consumption on specific metabolites to distinguish between MASLD and MetALD.

Figure S1 shows the design of the MR validation study. Data of alcohol consumption and metabolites were obtained from two large genome‐wide association studies (GWAS) in populations of European ancestry [20, 21]. Among them, alcohol consumption was defined based on the average intake of alcohol in units per week. The alcohol consumption study was adjusted for age, sex and age × sex interaction. The metabolites study was adjusted for age, sex, fasting status and a binary variable denoting the genotyping chip used in individuals (the UKBB Axiom array or the UK BiLEVE array). All data are available from the IEU summary database (Table S1). This study followed the Strengthening the Reporting of Observational Studies in Epidemiology using Mendelian Randomisation (STROBE‐MR) reporting guidelines [22] and the related checklist was provided (see STROBE‐ MR checklist). Ethical approval and written informed consent were obtained for all publicly available GWAS studies.

### 2.7 Instrumental Variables (IVs) Selection

A total of 39 single nucleotide polymorphisms (SNPs) highly correlated with alcohol consumption were obtained as IVs by setting a genome‐wide significance threshold (*p* < 5 × 10^−8^) and linkage disequilibrium parameters (*r* ^2^ < 0.01, clumping distance < 10,000 kb) (Table S2). We then evaluated the remaining SNPs' power using the *F* statistics $F\left(F=\frac{\left(N-2\right)\times R^{2}}{\left(1-R^{2}\right)}\right)$ for each SNP. Here, $R^{2}=\beta^{2}\times 2\times \mathrm{EAF}\times \left(1-\mathrm{EAF}\right)$, *N*, *β* and EAF indicates the sample size, beta value and effect allele frequency, respectively. SNPs with less statistical power would be removed (*F* statistics < 10). SNPs with inconsistent alleles between exposure and outcome (rs28601761) were excluded. Inconsistent alleles refer to cases where the allele representation does not match between the two datasets (e.g., A/G in the exposure dataset and G/A in the outcome dataset), which could result in incorrect alignment and introduce bias in the analysis. Additionally, SNPs associated with confounding factors such as smoking, BMI, abnormal liver function and diabetes (rs13107325, rs2049045, rs1229984, rs1260326, rs1229984) were excluded using the GWAS Catalog database. These rigorous selection procedures ensured the accuracy of the IVs and strictly adhered to the three core constructs of MR, namely the relevance assumption, the independence assumption and the exclusion restriction (Figure S1).

### 2.8 MR Analysis

Inverse variance weighted (IVW), MR‐Egger, weighted median, simple mode and weighted mode analyses were used to investigate the causal relationship between alcohol consumption and the metabolites. IVW, a widely used approach, integrates Wald ratios from multiple SNPs, provides unbiased estimates only when all IVs are valid and are sensitive to pleiotropic or confounded IVs. Conversely, the MR‐Egger regression incorporates intercepts and yields accurate estimates under the InSIDE assumption, even when accommodating horizontal pleiotropy [23]. The InSIDE assumption requires that the strength of instrumental variables, indicated by their associations with the exposure, is independent of their direct effects on the outcome. This allows MR‐Egger to address bias caused by unbalanced pleiotropy, a form of horizontal pleiotropy where genetic variants influence the outcome through pathways independent of the exposure, which could otherwise bias causal estimates. Meanwhile, the weighted median estimation method remains robust even when no more than 50% of IVs are invalid [24]. Notably, the weighted‐mode analysis offers advantages over MR‐Egger regression when the InSIDE assumption is not met [25].

The Cochran's Q test and MR‐Egger regression intercept analysis were conducted to test heterogeneity and pleiotropy of the MR results. The existence of heterogeneity and pleiotropy were determined by a *p* value < 0.05. Additionally, we performed MR pleiotropy residual sum and outlier (MR‐PRESSO) analyses to detect outliers defined by *p* value < 0.05 [26]. If the outliers were detected, they would be removed and we would reassess the MR causal estimation. The MR‐PRESSO‐corrected results are reported in the main results as well, as they adopted the IVW method. The leave one out method was conducted to test whether the MR results were driven by one single SNP.

All statistical analyses were performed using R version 4.0.3. Regression models were fitted using the ‘Im’ function from the ‘stats’ package. Graphics were created using the ‘ggplot2’ and ‘circos’ package. All MR analyses were conducted using ‘TwoSampleMR’ and ‘MRPRESSO’ packages. The flow chart was created with Microsoft PowerPoint Version 16.93, and the graphical abstract was created with biorender.com. All codes and scripts are maintained in a version‐controlled repository and are available upon request to the UK biobank repository to ensure the reproducibility of the analyses.

## 3 Results

### 3.1 Baseline Characteristics of MASLD and MetALD Patients

Of the SLD‐diagnosed participants with metabolomics and lipidomics data, 5539 MASLD participants and 462 MetALD participants were first analysed to see if their lipidomic profiles at baseline could distinguish MASLD from MetALD (Figure 1A). The first lipidomic measurements as well as the standard serum parameters were taken around 6 years prior to the liver MRI (Figure 1B). As we believe that the liver disease is slowly progressing and therefore quite stable over time, we first investigated differences in baseline serum profiles between MASLD and MetALD patients (Table 1). For liver enzymes, MetALD patients exhibited higher levels of serum alanine aminotransferases (ALT), aspartate aminotransferases (AST) and gamma‐glutamyl transferase (GGT) compared to the MASLD cohort (*p* < 0.05, Table 1). A similar trend was observed for Apolipoprotein A and HDL cholesterol, whereby MetALD participants had significantly higher levels compared to MASLD (HDL: 1.46 ± 0.36 mmol/L vs. 1.28 ± 03 mmol/L, *p* < 0.0001, Table 1).

**TABLE 1:** Summary statistics of patients with MetALD and MASLD.

Section	Metabolite (unit)	MetALD (mean ± SD) *n* = 644	MASLD (mean ± SD) *n* = 5426	Univariate *p*
Demographics at time of MRI				
Age (years)		54.15 ± 7.35	55.47 ± 7.31	**0.001**
BMI (kg/m^2^)		28.70 ± 3.92	29.20 ± 4.16	**0.009**
Female gender (%)		66 ± 47	61 ± 49	**0.041**
Liver lipid content on MRI (%)		11.81 ± 7	10.48 ± 5.81	**8E‐05**
Liver tests at baseline				
Alanine aminotransferase (U/L)		31.19 ± 17.13	29.37 ± 18.01	**0.033**
Albumin (g/L)		45.81 ± 2.45	45.52 ± 2.52	**0.022**
Alkaline phosphatase (U/L)		79.66 ± 22.74	84.06 ± 24.86	**0.0001**
Aspartate aminotransferase (U/L)		29.23 ± 10.90	27.75 ± 12.23	**0.007**
Direct bilirubin (μmol/L)		1.94 ± 0.74	1.85 ± 0.83	**0.018**
Total bilirubin (μmol/L)		9.79 ± 4.04	9.42 ± 4.82	0.07
Gamma glutamyltransferase (U/L)		57.73 ± 63.38	41.97 ± 36.13	**3E‐07**
Lipid panel at baseline				
Apolipoprotein A (g/L)		1.58 ± 0.26	1.44 ± 0.23	**8E‐21**
Apolipoprotein B (g/L)		1.09 ± 0.23	1.08 ± 0.24	0.34
Cholesterol (mmol/L)		5.93 ± 1.04	5.75 ± 1.13	**5E‐04**
HDL cholesterol (mmol/L)		1.46 ± 0.36	1.28 ± 0.3	**4E‐19**
LDL direct (mmol/L)		3.71 ± 0.81	3.68 ± 0.85	0.39
Lipoprotein A (mg/dL)		43.16 ± 49.21	48.67 ± 48.67	0.92
Triglycerides (mmol/L)		2.04 ± 1.14	2.15 ± 1.09	**0.043**
General biochemistry at baseline				
Calcium (mmol/L)		2.38 ± 0.09	2.38 ± 0.09	0.48
Creatinine (μmol/L)		74.54 ± 13.33	74.96 ± 14.07	0.52
Cystatin C (mg/L)		0.88 ± 0.12	0.91 ± 0.13	**2E‐06**
Glucose (mmol/L)		5.16 ± 1.01	5.14 ± 1.18	0.62
HbA1c (mmol/mol)		35.01 ± 5.27	36.24 ± 6.30	**5E‐06**
IGF‐1 (ng/mL)		20.71 ± 4.89	21.64 ± 5.51	**2E‐04**
Phosphate (mmol/L)		1.13 ± 0.17	1.14 ± 0.17	0.09
Urate (μmol/L)		356.78 ± 81.94	339.50 ± 75.02	**2E‐05**
Urea (mmol/L)		5.28 ± 1.21	5.42 ± 1.23	0.02
Vitamin D (nmol/L)		48.59 ± 22.48	46.39 ± 19.61	0.04
C‐reactive protein (mg/L)		2.33 ± 2.89	2.77 ± 3.73	**0.003**
Oestradiol (pmol/L)		387.03 ± 239.28	409.89 ± 329.26	0.44
Rheumatoid factor (IU/mL)		21.11 ± 14.35	22.95 ± 17.96	0.45
SHBG (nmol/L)		38.94 ± 18.66	38.37 ± 19.16	0.55
Testosterone (nmol/L)		8.38 ± 5.71	7.66 ± 5.56	**0.012**

*Note:* Comparisons were made between MASLD (allowing an alcohol consumption below 140 g/week for females and 210 g/week for males) and MetALD (MASLD + alcohol consumption between 140 to 350 g/week for females and 210 to 420 g/week for males). Continuous variables were expressed as means and standard deviations, while categorical variables were expressed as numbers and frequencies (%). Bold values indicate statistically significant values.

Abbreviations: HDL, high‐density lipoprotein; IGF‐1, Insulin growth factor‐1; LDL, low‐density lipoprotein; MASLD, metabolic dysfunction‐associated steatotic liver disease; MetALD, metabolic dysfunction and alcohol‐related liver disease; SHBG, sex‐hormone binding globulin.

### 3.2 Differential Expression of Lipidomic and Metabolomic Markers Between MASLD and MetALD

**FIGURE 2:**
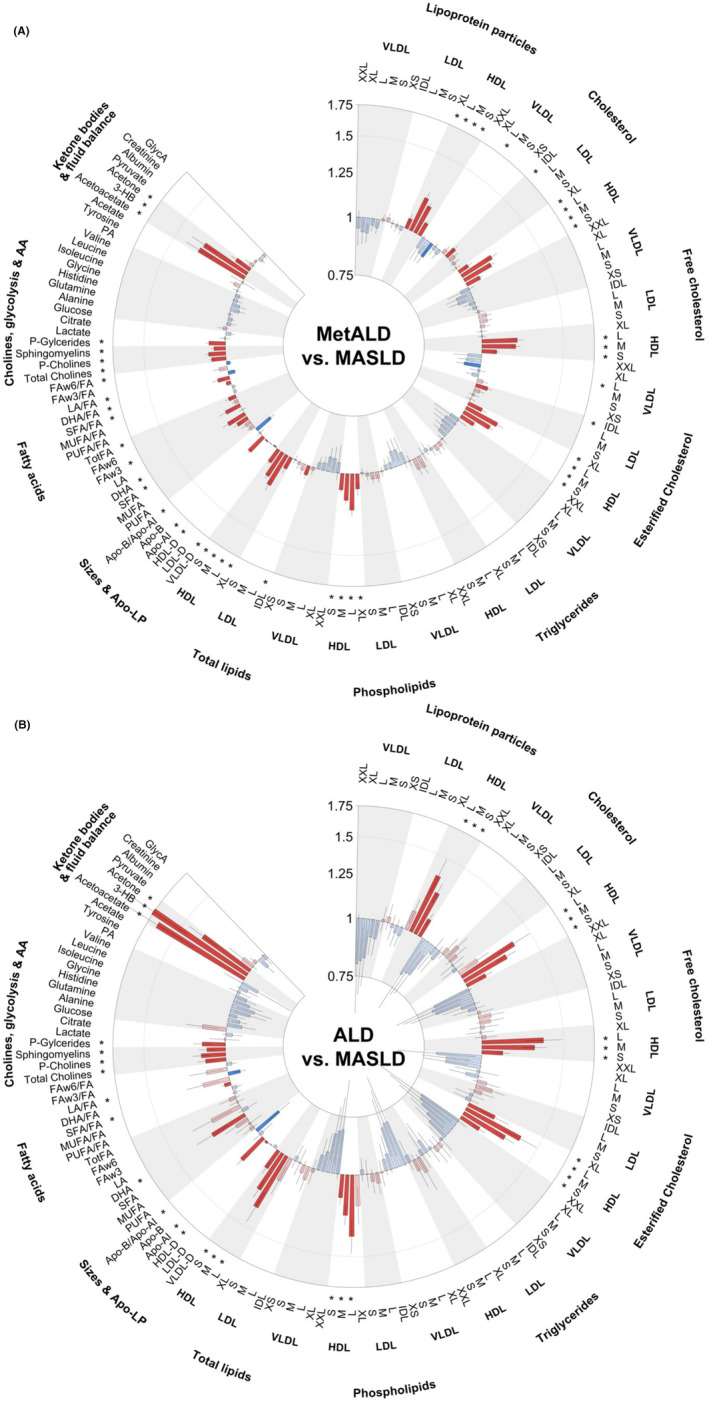
Circle plots for lipidomic analysis. (A) Circle plot for lipidomic analysis for MetALD versus MASLD UKB participants. Red = Higher in MetALD, blue = higher in MASLD. (B) Circle plot for lipidomic analysis for ALD versus MASLD UKB participants. Red = Higher in ALD, blue = higher in MASLD. Baseline Lipidomic parameters were measured through NMR spectroscopy. Hazard ratios (with 95% confidence intervals) are presented per 1‐SD higher metabolic biomarker on the natural log scale, stratified by age, sex, body mass index and Townsend deprivation index. *Original code by Diego J. Aguilar‐Ramirez. Apo‐A1, apolipoprotein A1, Apo‐B, apolipoprotein B, FA, fatty acids, FAw3, omega‐3 fatty acids, FAw6, omega‐6 fatty acids, LA, linoleic acid, MUFA, monounsaturated fatty acids, PUFA, polyunsaturated fatty acids, SFA, saturated fatty acids, TG, triglycerides, 3‐HB, 3‐Hydroxybutyrate, DHA, docosahexaenoic acid.

The unbiased analysis included 249 lipidomic and metabolomic parameters, which were evaluated for their potential to discriminate between MASLD and MetALD. After adjusting for age, sex and BMI, we identified a significant difference in the lipidomic profiles of MASLD and MetALD. There are 107 differentially expressed lipidomic markers when comparing MetALD to MASLD patients (Figure 2A). Of lipid parameters, large‐sized VLDL lipoprotein was significantly lower in MetALD participants. Consistently, total HDL lipids were significantly higher, with the largest effect ize distinguishing MetALD and MASLD among lipidomic parameters (Table 2).

**TABLE 2:** Top 30 discriminatory metabolites between MetALD and MASLD.

Metabolite	OR	95% CI		*p*
Acetoacetate	1.30	1.21	1.41	1.48E‐11
3‐hydroxybutyrate	1.29	1.20	1.39	1.72E‐11
Phospholipids in large HDL	1.20	1.16	1.23	1.85E‐32
Concentration of large HDL particles	1.20	1.16	1.24	6.76E‐26
Free cholesterol in large HDL	1.19	1.15	1.23	1.86E‐24
Cholesterol in large HDL	1.19	1.15	1.23	7.54E‐24
Cholesteryl esters in large HDL	1.19	1.15	1.23	2.38E‐23
Total lipids in large HDL	1.19	1.15	1.22	1.77E‐28
Free cholesterol in medium HDL	1.18	1.16	1.21	3.08E‐50
Cholesterol in medium HDL	1.17	1.14	1.19	3.08E‐52
Cholesteryl esters in medium HDL	1.16	1.14	1.18	7.23E‐52
Concentration of medium HDL particles	1.16	1.14	1.18	9.25E‐51
Total lipids in medium HDL	1.15	1.13	1.17	6.12E‐51
Phospholipids in medium HDL	1.14	1.12	1.16	3.88E‐49
Apolipoprotein A1	1.10	1.09	1.12	3.82E‐49
Phosphatidylcholines	1.09	1.07	1.10	5.53E‐27
Phospholipids in small HDL	1.09	1.07	1.10	1.86E‐36
Acetone	1.08	1.04	1.12	3.25E‐9
Phosphoglycerides	1.08	1.07	1.10	3.09E‐25
Total lipids in small HDL	1.08	1.06	1.09	4.9E‐33
Cholesteryl esters in small HDL	1.08	1.06	1.09	4.16E‐29
Cholesterol in small HDL	1.08	1.06	1.09	5.74E‐31
Free cholesterol in small HDL	1.08	1.06	1.09	7.56E‐28
Total cholines	1.07	1.06	1.09	5.93E‐24
Concentration of small HDL particles	1.07	1.06	1.08	3.22E‐28
Sphingomyelins	1.06	1.04	1.07	1.02E‐15
Saturated fatty acids to total fatty acids percentage	1.02	1.02	1.03	8.09E‐15
Average diameter for HDL particles	1.01	1.00	1.01	6.15E‐16
Linoleic acid to total fatty acids percentage	0.97	0.96	0.98	3.91E‐10
Apolipoprotein B to apolipoprotein A1 ratio	0.91	0.89	0.94	2.62E‐12

*Note:* Differential expression of baseline lipidomic markers between MASLD and MetALD was determined using linear regression models corrected for age, sex and BMI. Metabolites with the highest discriminatory power between the two conditions were identified based on their OR, *p*‐values and 95% confidence intervals. A rank list of the top 30 metabolites with the lowest *p*‐values was generated for further investigation and sorted by OR.

Abbreviations: HDL, high‐density lipoprotein; MASLD, metabolic dysfunction‐associated steatotic liver disease; MetALD, metabolic dysfunction‐associated alcohol‐related liver disease.

### 3.3 Top 30 Discriminatory Metabolites in MetALD

The top metabolites with the highest discriminatory power were identified (Table 2). These included: Acetoacetate (OR: 1.30, 95% CI: 1.21–1.41; *p* = 1,48E‐11), 3‐Hydroxybutyrate (OR:1.29, 95% CI: 1.20–1.39; *p* = 1,72E‐11), Phospholipids in Large HDL (OR: 1.20, 95% CI: 1.16–1.23; *p* = 1,85E‐32), Concentration of large HDL particles, free cholesterol in large HDL, cholesterol in large HDL, cholesteryl esters in large HDL, Total lipids in large HDL, free cholesterol in medium HDL and cholesterol in medium HDL. All of these were significantly higher in the MetALD group compared to the MASLD group. Strikingly, the metabolites with the greatest effect sizes to differentiate MASLD and MetALD are associated with large‐sized HDL particles such as the concentration, total lipids, cholesterol and phospholipids in large HDL. To analyse the nature of the relationship of HDL and Acetoacetate with the risk of MetALD, we constructed cubic splines (Figures S2 and S3) and saw a nearly linear relationship. To account for the effect of potential differences in cT1 levels, we corrected the analyses for cT1 and saw comparable results (Figure S4). Next, we analysed the predictive capability of the lipids using AUROC; here again, Medium HDL‐related parameters performed best and even better than cT1 or Age (Table S3).

### 3.4 Sensitivity Analyses With ALD Patients

As a sensitivity analysis, we additionally compared MASLD to 53 cases of patients with proven ALD (Table 3). Interestingly, the majority of ALD participants were male and exhibited a significantly higher percentage of liver lipid content on MRI compared to MASLD. AST was statistically higher in ALD, while alkaline phosphatase was lower compared to MASLD. GGT levels were nearly doubled in ALD participants compared to MASLD patients (76.17 ± 56.38 U/L vs. 41.97 U/L ± 36.13, *p* = 1.19E‐04, Table 3).

**TABLE 3:** Summary statistics of patients with ALD and MASLD.

Section	Metabolite (unit)	ALD (mean ± SD) *n* = 53	MASLD (mean ± SD) *n* = 5423	Univariate *p*
Demographics at time of liver MRI				
Age (years)		55.42 ± 6.23	55.47 ± 7.31	0.95
BMI (kg/m^2^)		27.95 ± 3.95	29.20 ± 4.16	0.02
Female gender		0.74 ± 0.45	0.61 ± 0.49	**0.047**
Liver lipid content on MRI (%)		14.77 ± 9.1	10.48 ± 5.81	**0.001**
Liver tests				
Alanine aminotransferase (U/L)		31.41 ± 18.66	29.38 ± 18.01	0.46
Albumin (g/L)		46.26 ± 2.55	45.52 ± 2.52	0.06
Alkaline phosphatase (U/L)		76.14 ± 20.58	84.06 ± 24.86	**0.011**
Aspartate aminotransferase (U/L)		33.26 ± 13.87	27.75 ± 12.23	**0.009**
Direct bilirubin (μmol/L)		2.11 ± 0.81	1.85 ± 0.83	**0.04**
Total bilirubin (μmol/L)		9.90 ± 4.53	9.42 ± 4.82	0.47
Gamma glutamyltransferase (U/L)		76.17 ± 56.38	41.97 ± 36.13	**1.19E‐04**
Lipid panel at baseline				
Apolipoprotein A (g/L)		1.66 ± 0.36	1.44 ± 0.23	**0.00024**
Apolipoprotein B (g/L)		1.05 ± 0.24	1.08 ± 0.24	0.36
Cholesterol (mmol/L)		5.96 ± 1.21	5.75 ± 1.13	0.23
HDL cholesterol (mmol/L)		1.57 ± 0.42	1.28 ± 0.3	**4.56E‐05**
LDL direct (mmol/L)		3.64 ± 0.92	3.68 ± 0.85	0.80
Lipoprotein A (mg/dL)		33.97 ± 38.60.74	42.87 ± 48.67	0.19
Triglycerides (mmol/L)		1.94 ± 1.08	2.15 ± 1.09	0.18
General biochemistry at baseline				
Calcium (mmol/L)		2.40 ± 0.09	2.38 ± 0.09	0.10
Creatinine (μmol/L)		76.31 ± 16.03	74.96 ± 14.07	0.56
Cystatin C (mg/L)		0.86 ± 0.12	0.91 ± 0.13	0.**009**
Glucose (mmol/L)		5.63 ± 2.16	5.14 ± 1.18	0.14
HbA1c (mmol/mol)		36.17 ± 7.64	36.24 ± 6.30	0.95
IGF‐1 (ng/mL)		19.81 ± 5.17	21.64 ± 5.51	**0.02**
Phosphate (mmol/L)		1.10 ± 0.16	1.14 ± 0.17	0.10
Urate (μmol/L)		371.11 ± 87.69	339.50 ± 75.02	**0.016**
Urea (mmol/L)		5.29 ± 1.28	5.42 ± 1.23	0.49
Vitamin D (nmol/L)		46.42 ± 23.6	46.29 ± 19.61	0.97
C‐reactive protein (mg/L)		1.52 ± 1.42	2.77 ± 3.73	**2E‐07**
Oestradiol (pmol/L)		276.9 ± 136.74	409.89 ± 329.27	0.15
Rheumatoid factor (IU/mL)		26.33 ± 31.78	22.95 ± 17.96	0.85
SHBG (nmol/L)		36.75 ± 17.64	38.37 ± 19.16	0.54
Testosterone (nmol/L)		8.58 ± 5.52	7.62 ± 5.56	0.26

*Note:* Comparisons were made between MASLD (with either no or alcohol consumption below 140 g/week for females and 210 g/week for males) and ALD (defined as alcohol consumption above 350 g/week for females and 420 g/week for males). Continuous variables were expressed as means and standard deviations, while categorical variables were expressed as numbers and frequencies (%).

Abbreviations: ALD, alcohol‐related liver disease; HDL, high density lipoprotein; IGF‐1, insulin growth factor‐1; LDL, low density lipoprotein; MASLD, metabolic dysfunction‐associated steatotic liver disease; SHBG, sex‐hormone binding globulin.

Lipidomic profiling revealed 32 metabolites that were differentially expressed in ALD participants compared to MASLD (Figure 2B). Of these, large‐, medium‐ and small‐sized particles of HDL as well as HDL cholesterol and phospholipids were significantly higher in the ALD cohort. Apolipoprotein A1, saturated fatty acids and total choline were also overrepresented in ALD participants.

Similar to the top discriminatory metabolites identified in MetALD, participants with ALD also exhibited the highest discriminatory power with phospholipids, total lipids, concentration and cholesterol in large HDL, followed by apolipoprotein A1 (Table 4). Intriguingly, the effect size was highest for 3‐hydroxybutyrate (OR: 1.76, 95% CI: 1.44–2.15; *p* = 4,01E‐08) and acetoacetate (OR:1.66, 95% CI: 1.35–2.06; *p* = 2,2E‐06). For both ALD and MetALD, large HDL components (Cholesterol, Cholesteryl Esters, Phospholipids and Total Lipids) show elevated levels. However, ALD shows even higher ORs (ranging from 1.34 to 1.36) compared to MetALD (ranging from 1.19 to 1.20, Table 4). The significance is profound in both groups, but the effect sizes in ALD are higher. Regarding Apolipoprotein A1, ALD shows a much higher OR (1.16, 95% CI: 1.12–1.21; *p* = 2.79E‐16, Table 4) compared to MetALD (1.10, 95% CI: 1.09–1.12; *p* = 3.82E‐49, Table 2), again suggesting that apolipoprotein A1 alterations are more pronounced in ALD.

**TABLE 4:** Top 30 discriminatory metabolites between ALD and MASLD.

Metabolite	OR	95% CI		*p*
3‐hydroxybutyrate	1.76	1.44	2.15	4.01E‐08
Acetoacetate	1.66	1.35	2.06	2.2E‐06
Concentration of large HDL particles	1.36	1.24	1.49	7.89E‐11
Cholesteryl esters in large HDL	1.36	1.24	1.50	3.84E‐10
Cholesterol in large HDL	1.36	1.24	1.49	2.55E‐10
Free cholesterol in large HDL	1.36	1.24	1.49	1.85E‐10
Phospholipids in large HDL	1.36	1.25	1.47	4.47E‐13
Total lipids in large HDL	1.34	1.23	1.46	8.87E‐12
Acetone	1.33	1.22	1.46	1.33E‐10
Free cholesterol in medium HDL	1.30	1.22	1.38	5.38E‐17
Cholesterol in medium HDL	1.27	1.21	1.35	4.03E‐18
Cholesteryl esters in medium HDL	1.27	1.20	1.34	4.22E‐18
Concentration of medium HDL particles	1.26	1.19	1.33	5.32E‐17
Total lipids in medium HDL	1.24	1.18	1.30	6.42E‐17
Phospholipids in medium HDL	1.22	1.17	1.28	5.83E‐16
Docosahexaenoic acid	1.21	1.10	1.33	4.99E‐05
Apolipoprotein A1	1.16	1.12	1.21	2.79E‐16
Phosphatidylcholines	1.13	1.08	1.18	5.08E‐08
Phospholipids in small HDL	1.12	1.08	1.16	2.14E‐10
Phosphoglycerides	1.12	1.07	1.17	2.01E‐07
Cholesteryl esters in small HDL	1.11	1.07	1.16	9.3E‐09
Cholesterol in small HDL	1.11	1.07	1.15	3.34E‐09
Total lipids in small HDL	1.11	1.07	1.15	2.39E‐09
Free cholesterol in small HDL	1.11	1.07	1.15	3.49E‐08
Total cholines	1.11	1.06	1.15	3.72E‐07
Concentration of small HDL particles	1.10	1.06	1.14	4.22E‐08
Sphingomyelins	1.10	1.05	1.14	4.65E‐06
Average diameter for HDL particles	1.01	1.01	1.01	2.42E‐07
Linoleic acid to total fatty acids percentage	0.94	0.91	0.97	5.01E‐05
Apolipoprotein B to apolipoprotein A1 ratio	0.86	0.80	0.93	7.24E‐05

*Note:* Differential expression of baseline lipidomic markers between MASLD and MetALD was determined using linear regression models corrected for age, sex and BMI. Metabolites with the highest discriminatory power between the two conditions were identified based on their OR, *p*‐values and 95% confidence intervals. A rank list of the top 30 metabolites with the lowest *p*‐values was generated for further investigation and sorted by OR.

Abbreviations: HDL, high‐density lipoprotein; MASLD, metabolic dysfunction‐associated steatotic liver disease; MetALD, metabolic dysfunction and alcohol‐related liver disease.

### 3.5 Time‐Dependent Validation of Large HDL and Acetoacetate as Markers of Alcohol‐Related Liver Disease

At the first follow‐up, in a small subset of patients, the lipidomic analyses were repeated, around 4 years after the initial examinations and 2 years prior to the liver MRI (Figure 1B). Here, 1154 MASLD patients, 97 MetALD and 11 ALD patients were included. To prove that the lipidomic signature is stable over time, we first correlated the levels of Acetoacetate and Phospholipids in Large HDL and found that Acetoacetate was an unstable parameter over time (*R* ^2^ = 0.11), but HDL‐related parameters were very stable (*R* ^2^ = 0.83, Figures S5 and S6). Even though only a minority of patients had measurements, we repeated the analyses comparing MetALD and MASLD, as well as ALD and MASLD. Strikingly, the HDL phenotype, especially concerning large HDL‐associated parameters, was repeated, but Acetoacetate was not significantly different in MetALD versus MASLD, but in ALD vs. MASLD (Figures S7 and S8).

Lastly, we performed an enrichment analysis of metabolite and lipid pathways in MetaboAnalyst and found that differentially expressed metabolites from MetALD cases in the UKB are consistent with pathways related to ketone body metabolism and fatty acid biosynthesis (Figure S9, Table 4).

### 3.6 Genetic Level Validation of Causal Association Between Alcohol Consumption and Top 30 Discriminatory Metabolites Using MR Analysis

**FIGURE 3:**
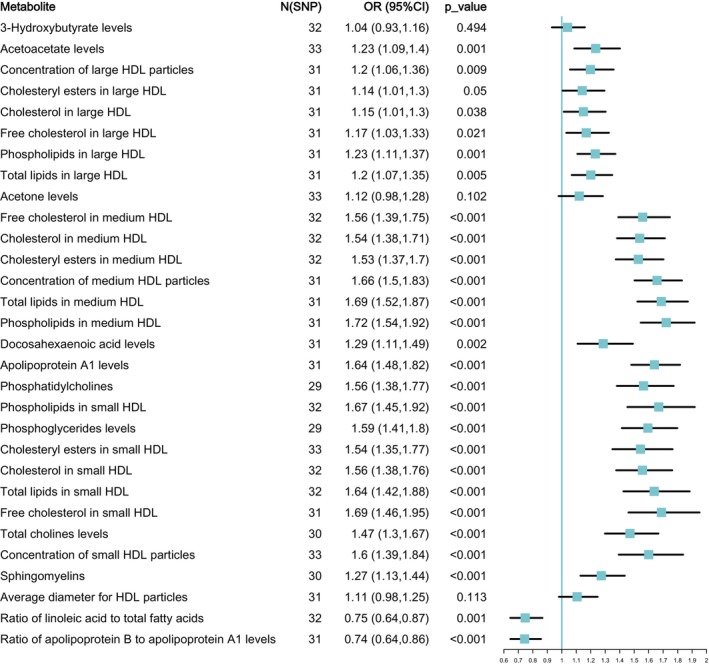
Forest Plot of Mendelian randomisation analysis between alcohol consumption and the top 30 discriminatory metabolites using Inverse variance weighting. OR, odds ratio; 95% CI, 95% confidence interval.

To validate the association of HDL with alcohol‐related liver phenotypes, we used MR analysis. In our MR analysis, genetically‐predicted alcohol consumption was associated with increased levels of HDL‐related metabolites (Table S5, Figure 3). These included Acetoacetate levels (OR_IVW_: 1.23, 95% CI: 1.09–1.4; *p* = 0.001), Concentration of Large HDL Particles (OR_IVW_: 1.2, 95% CI: 1.06–1.36; *p* = 0.009) and Total Lipids in Medium HDL (OR_IVW_: 1.69, 95% CI: 1.52–1.87; *p* < 0.001).

There were pleiotropic effects for acetoacetate levels, as some genetic variants affected outcomes through pathways independent of the target exposure, thereby potentially biasing the MR results. Nevertheless, the MR‐Egger and weighted median methods consistently supported a causal relationship (Tables S5–S8). Furthermore, the ‘leave‐one‐out’ method, which systematically excluded one single SNP at a time from the analysis, was used to assess the robustness of the findings. This approach confirmed that the observed results were not unduly influenced by any individual SNP, thereby reinforcing the robustness of the analysis (Figure S10A–E).

## 4 Discussion

The results of this study reveal the plasma lipidomic differences between MASLD and MetALD, offering potential avenues for diagnostic tools. Particularly, HDL‐centric markers were significantly overrepresented in MetALD and ALD cases, thereby suggesting these markers might highlight pathophysiologically relevant differences in altered lipoprotein metabolism in alcohol‐related steatotic liver disease compared to MASLD. Previous studies have outlined that ethanol metabolism influences HDL concentrations through the enzymatic action of alcohol dehydrogenase and the subsequent production of acetaldehyde [27, 28]. The specific mechanism by which alcohol can increase HDL lipids is not entirely clear, but may be attributed to the role of HDL in the reverse cholesterol transport (RCT) system [29]. Moderate alcohol intake has been extensively studied in relation to atherosclerotic risk [30], with pioneering studies hypothesising that alcohol consumption raises HDL cholesterol due to the upregulation of transport rates of HDL apolipoproteins A1 and A2 [31]. Cellular cholesterol efflux is implicated in the early stages of RCT, which has been proposed to work by resorbing cholesterol to phospholipid‐containing acceptors such as apolipoproteins A1 in HDL particles [32].

Apolipoprotein A1, the principal protein component of HDL, exhibited elevated levels in MetALD. The hypothesis is that the RCT system is potentially upregulated in MetALD participants as well, a phenomenon that may be influenced by the interference of ethanol with apolipoprotein metabolism. Phospholipids in various HDL subfractions were also noted as discriminative features between MASLD and MetALD. These findings are supported by previous studies identifying plasma levels of HDL phospholipids to be major determinants of cholesterol efflux, which are increased after alcohol consumption [33, 34]. Given their pivotal role in lipoprotein structure and cholesterol transport, these alterations could arise from alcohol consumption and support the impact of alcohol on lipid metabolism, extending specifically to HDL subfractions [35]. Generally, MetALD and ALD phenotypes exhibited higher large and medium‐sized HDL particles, which reflect previous studies demonstrating a favourable shift towards larger HDL particles with a dose‐dependent increase with alcohol intake [36, 37]. This may be due to the influence of alcohol intake on increased lipoprotein lipase activity while downregulating hepatic lipase and cholesteryl ester transfer protein (CETP) activity, which would be expected to cause an increase in HDL particle sizes [31, 38, 39, 40, 41]. A combination of catabolic changes and apolipoprotein production may be responsible for the observed differences in HDL particle size, but further studies are needed.

Our study highlights the lipidomic distinctiveness between MASLD and MetALD, but these findings need to be compared to the existing body of literature that aims to distinguish MASLD from MetALD and ALD [42]. Notably, both ALD and MetALD displayed pronounced changes in Medium, Small and Large HDL particle constituents compared to MASLD. However, ALD demonstrated higher odds ratios across these categories, indicative of more severe metabolic disruption [43]. Our paper points in the direction that the introduction of the new category MetALD was justified, as the metabolome/lipidome resembles ALD better than MASLD but still differs from ALD.

Our findings highlight the importance of considering the disease‐specific metabolic landscape in ALD, MetALD and MASLD for both diagnostic and potential therapeutic purposes. This is primarily due to the pathological overlap attributed to lipid metabolism across these three disease processes. *De novo* lipogenesis, fatty acid oxidation and cholesterol transport systems play a crucial role in the pathogenesis of MASLD, with a particular focus on LDL and VLDL production in hepatocytes [44]. Alterations in VLDL regulation are also implicated by phospholipid homeostasis, whereby phosphatidylcholine is a major component that aids in the assembly and secretion of VLDL in MASLD [45]. Meanwhile, experimental models suggest that the degree of lipid dysregulation is exacerbated in the presence of alcohol consumption via AMP‐activated protein kinase, which contributes to the effect size of lipid aberrations in the metabolomics of MetALD and ALD participants compared to MASLD [46]. The most recent lipidomic profiling of ALD and MASLD suggested the role of alcohol in increasing the uptake of free fatty acids and lysophosphatidylcholine, inducing lipotoxicity and accelerating liver injury [14, 47]. What differentiates our findings from former research is the particular emphasis on medium HDL subfractions as opposed to markers in VLDL, LDL and fatty acid parameters. Though the effect sizes are greatest in ALD, the presence of moderate drinking alone has been shown to potentially elevate HDL by about 3.8 mg/dL [37]. The linear association between alcohol intake and HDL among heavy drinkers compared to non‐drinkers is well‐supported by former studies, even in the presence of insulin resistance, suggesting the importance of various HDL subfractions as a key discriminator between MASLD and MetALD [48]. Changes in HDL size and composition, and therefore function, have been implicated in multiple factors of metabolic disease pathophysiology [49]. Additional investigations into the role of HDL in MetALD and MASLD could provide valuable information on mechanistic differences between the two diseases.

This study is not without its limitations that need to be acknowledged for a comprehensive interpretation of the findings. A key constraint was the unavailability of lipidomic measurements at the day of the liver MRI assessment. Metabolomics data were collected at baseline and follow‐up 1, while the MRI was performed at a follow‐up 2 visit, which poses a limitation in evaluating the temporal relationship between ongoing alcohol intake and the observed lipidomic changes. We tried to account for this by analysing both available timepoints to show that the HDL phenotype is stable over time. Moreover, the study uses data from the UK Biobank, which has its own set of limitations. The cohort is predominantly of European ancestry, thereby potentially limiting the generalisability of the findings to more ethnically diverse populations. The voluntary nature of participation in the UK Biobank might also introduce a ‘healthy volunteer effect’, as individuals who choose to participate tend to be healthier than the general population, thus potentially attenuating the observed disease associations. The cross‐sectional design inherent in much of the UK Biobank data also precludes drawing causal inferences from the observed associations. While our mean‐based normalisation approach facilitates comparisons across metabolites, it does not account for potential non‐linear relationships or differences in variance. Future studies could consider alternative methods, such as rank‐based inverse normal transformation. Additionally, In MR analysis, alcohol consumption was based on self‐reported data, which may be subject to subjective bias and fail to adequately capture the diversity of drinking patterns (e.g., frequency, intensity and episodic drinking). These limitations reduce the accuracy and comprehensiveness of assessing its associations with specific metabolites. Although studies have selected SNPs significantly associated with alcohol consumption based on GWAS data, these variants may lack a biological basis that directly reflects alcohol consumption [50].

In conclusion, our data show the lipidomic dissimilarity of MASLD, MetALD and ALD, emphasising the necessity for disease‐specific metabolic assessments. Considering the higher mortality rate of patients suffering from MetALD [9], our findings offer possibilities to identify individuals earlier, enabling timely and targeted treatment. These insights could pave the way for targeted interventions that consider the unique lipidomic signatures.

## Author Contributions

**Kai Markus Schneider:** conceptualization, funding acquisition, writing – original draft, methodology, supervision, writing – review and editing, project administration. **Feng Cao:** investigation, formal analysis, resources, writing – review and editing, visualization. **Helen Ye Rim Huang:** conceptualization, visualization, writing – original draft. **Lanlan Chen:** conceptualization, supervision, validation, formal analysis. **Yazhou Chen:** supervision, validation, formal analysis. **Rongpeng Gong:** validation, supervision, formal analysis. **Anastasia Raptis:** writing – review and editing, project administration, validation. **Kate Townsend Creasy:** writing – review and editing. **Jan Clusmann:** writing – review and editing, software. **Felix van Haag:** writing – review and editing. **Paul‐Henry Koop:** writing – review and editing. **Adrien Guillot:** conceptualization, writing – review and editing, supervision. **Tom Luedde:** conceptualization, writing – review and editing. **Rohit Loomba:** conceptualization, writing – original draft, resources. **Sven Francque:** writing – original draft, conceptualization, resources. **Carolin Victoria Schneider:** conceptualization, investigation, formal analysis, data curation, software, methodology, visualization, funding acquisition, writing – original draft, writing – review and editing, project administration, resources.

## Conflicts of Interest

RL serves as a consultant to Aardvark Therapeutics, Altimmune, Arrowhead Pharmaceuticals, AstraZeneca, Cascade Pharmaceuticals, Eli Lilly, Gilead, Glympse bio, Inipharma, Intercept, Inventiva, Ionis, Janssen Inc., Lipidio, Madrigal, Neurobo, Novo Nordisk, Merck, Pfizer, Sagimet, 89 bio, Takeda, Terns Pharmaceuticals and Viking Therapeutics. In addition, his institution received research grants from Arrowhead Pharmaceuticals, Astrazeneca, Boehringer‐Ingelheim, Bristol‐Myers Squibb, Eli Lilly, Galectin Therapeutics, Gilead, Intercept, Hanmi, Intercept, Inventiva, Ionis, Janssen, Madrigal Pharmaceuticals, Merck, Novo Nordisk, Pfizer, Sonic Incytes and Terns Pharmaceuticals. He is a co‐founder of LipoNexus Inc.

## Supporting information


Data S1.

## Data Availability

The data that support the findings of this study are available on request from the corresponding author. The data are not publicly available due to privacy or ethical restrictions.
