# Supplementary material for: The Lipidomic Profile Discriminates Between MASLD and MetALD
Source: Aliment Pharmacol Ther. 2025 Feb 11;61(8):1357–71. doi: 10.1111/apt.70012 (PMC11950808; doi:10.1111/apt.70012)
Supplement: Supplementary file 1 — Data S1. [file APT-61-1357-s001.docx]

**Supplement of The Lipidomic Profile discriminates between metabolic dysfunction-associated steatotic liver disease and combined metabolic dysfunction-and alcohol-related steatotic liver disease**

**Supplemental table 1: Mendelian Randomization study data of the top 30 discriminatory metabolites source.**

| **GWAS ID** | **Trait** | **Year** | **Sample size** | **Number of SNPs** |
| --- | --- | --- | --- | --- |
| ebi-a-GCST90092811 | 3-Hydroxybutyrate levels | 2022 | 113594 | 11590399 |
| ebi-a-GCST90092804 | Acetoacetate levels | 2022 | 115079 | 11590399 |
| ebi-a-GCST90092851 | Concentration of large HDL particles | 2022 | 115082 | 11590399 |
| met-d-L_HDL_CE | Cholesteryl esters in large HDL | 2020 | 115078 | 12321875 |
| met-d-L_HDL_C | Cholesterol in large HDL | 2020 | 115078 | 12321875 |
| met-d-L_HDL_FC | Free cholesterol in large HDL | 2020 | 115078 | 12321875 |
| met-d-L_HDL_PL | Phospholipids in large HDL | 2020 | 115078 | 12321875 |
| met-d-L_HDL_L | Total lipids in large HDL | 2020 | 115078 | 12321875 |
| ebi-a-GCST90092805 | Acetone levels | 2022 | 115079 | 11590399 |
| met-d-M_HDL_FC | Free cholesterol in medium HDL | 2020 | 115078 | 12321875 |
| met-d-M_HDL_C | Cholesterol in medium HDL | 2020 | 115078 | 12321875 |
| met-d-M_HDL_CE | Cholesteryl esters in medium HDL | 2020 | 115078 | 12321875 |
| ebi-a-GCST90092899 | Concentration of medium HDL particles | 2022 | 115082 | 11590399 |
| met-d-M_HDL_L | Total lipids in medium HDL | 2020 | 115078 | 12321875 |
| met-d-M_HDL_PL | Phospholipids in medium HDL | 2020 | 115078 | 12321875 |
| ebi-a-GCST90092816 | Docosahexaenoic acid levels | 2022 | 115006 | 11590399 |
| ebi-a-GCST90092808 | Apolipoprotein A1 levels | 2022 | 115082 | 11590399 |
| met-d-Phosphatidylc | Phosphatidylcholines | 2020 | 114999 | 12321875 |
| met-d-S_HDL_PL | Phospholipids in small HDL | 2020 | 115078 | 12321875 |
| ebi-a-GCST90092938 | Phosphoglycerides levels | 2022 | 115006 | 11590399 |
| met-d-S_HDL_CE | Cholesteryl esters in small HDL | 2020 | 115078 | 12321875 |
| met-d-S_HDL_C | Cholesterol in small HDL | 2020 | 115078 | 12321875 |
| met-d-S_HDL_L | Total lipids in small HDL | 2020 | 115078 | 12321875 |
| met-d-S_HDL_FC | Free cholesterol in small HDL | 2020 | 115078 | 12321875 |
| ebi-a-GCST90092812 | Total cholines levels | 2022 | 115006 | 11590399 |
| ebi-a-GCST90092951 | Concentration of small HDL particles | 2022 | 115082 | 11590399 |
| met-d-Sphingomyelins | Sphingomyelins | 2020 | 114999 | 12321875 |
| ebi-a-GCST90092828 | Average diameter for HDL particles | 2022 | 115082 | 11590399 |
| ebi-a-GCST90092881 | Ratio of linoleic acid to total fatty acids | 2022 | 115006 | 11590399 |
| ebi-a-GCST90092810 | Ratio of apolipoprotein B to apolipoprotein A1 levels | 2022 | 115082 | 11590399 |

**Supplemental table 2: The information of single nucleotide polymorphism data of alcohol consumption.**

| **SNP** | **Chromosome** | **EA** | **OA** | **Beta_value** | **Se_value** | **P_value** | **F_value** |
| --- | --- | --- | --- | --- | --- | --- | --- |
| rs1004787 | 2 | A | G | 1.53E-02 | 1.94E-03 | 3.31E-15 | 59.83 |
| rs10085696 | 7 | G | A | -1.61E-02 | 2.49E-03 | 1.24E-10 | 44.28 |
| rs11860773 | 16 | C | T | -1.50E-02 | 2.44E-03 | 8.35E-10 | 33.66 |
| rs1229984 | 4 | C | T | 1.88E-01 | 6.18E-03 | 1.00E-200 | 1636.50 |
| rs1260326 | 2 | C | T | 2.38E-02 | 1.98E-03 | 3.33E-33 | 145.51 |
| rs13024996 | 2 | A | C | -1.20E-02 | 2.00E-03 | 2.08E-09 | 35.04 |
| rs1302808 | 4 | A | C | 2.39E-02 | 2.39E-03 | 1.36E-23 | 88.96 |
| rs13107325 | 4 | T | C | -3.65E-02 | 3.91E-03 | 1.23E-20 | 85.82 |
| rs13332432 | 16 | G | C | 1.40E-02 | 2.14E-03 | 5.94E-11 | 42.26 |
| rs1387766 | 12 | A | G | -1.08E-02 | 1.98E-03 | 4.79E-08 | 29.50 |
| rs153106 | 16 | C | T | -1.36E-02 | 1.96E-03 | 3.63E-12 | 47.62 |
| rs16854020 | 4 | A | G | 1.81E-02 | 2.91E-03 | 4.82E-10 | 38.69 |
| rs17542254 | 11 | G | A | 1.31E-02 | 2.15E-03 | 8.96E-10 | 34.67 |
| rs2049045 | 11 | C | G | -1.38E-02 | 2.51E-03 | 3.97E-08 | 30.96 |
| rs2299409 | 7 | A | G | -1.06E-02 | 1.93E-03 | 4.80E-08 | 29.75 |
| rs28601761 | 8 | G | C | 1.13E-02 | 1.96E-03 | 7.60E-09 | 32.18 |
| rs28680958 | 1 | A | G | -1.36E-02 | 2.37E-03 | 9.78E-09 | 34.78 |
| rs28712821 | 4 | A | G | 2.83E-02 | 1.97E-03 | 1.10E-46 | 203.61 |
| rs28732378 | 3 | G | A | -1.67E-02 | 2.19E-03 | 2.24E-14 | 58.60 |
| rs28929474 | 14 | T | C | -4.77E-02 | 7.14E-03 | 2.39E-11 | 36.43 |
| rs331939 | 4 | A | G | -1.19E-02 | 2.03E-03 | 4.50E-09 | 34.07 |
| rs34121753 | 17 | G | A | 1.11E-02 | 1.95E-03 | 1.39E-08 | 31.62 |
| rs3814877 | 16 | T | G | -1.13E-02 | 1.97E-03 | 1.07E-08 | 31.62 |
| rs4309187 | 11 | C | A | 1.48E-02 | 2.09E-03 | 1.37E-12 | 49.08 |
| rs4752999 | 11 | T | C | -1.46E-02 | 2.07E-03 | 2.03E-12 | 48.95 |
| rs55872084 | 5 | T | G | 1.27E-02 | 2.27E-03 | 1.98E-08 | 29.27 |
| rs55932213 | 9 | G | A | 1.25E-02 | 2.22E-03 | 1.80E-08 | 33.67 |
| rs6106989 | 20 | A | G | 1.09E-02 | 1.98E-03 | 3.81E-08 | 28.90 |
| rs6573197 | 14 | T | C | -1.23E-02 | 2.14E-03 | 9.30E-09 | 31.89 |
| rs6739804 | 2 | C | T | -1.30E-02 | 2.08E-03 | 4.72E-10 | 40.18 |
| rs676388 | 19 | C | T | 1.51E-02 | 1.93E-03 | 5.49E-15 | 60.63 |
| rs682011 | 11 | C | T | 1.10E-02 | 1.95E-03 | 1.53E-08 | 31.69 |
| rs6969458 | 7 | A | G | 1.27E-02 | 1.94E-03 | 5.20E-11 | 40.87 |
| rs7106546 | 11 | T | G | 1.30E-02 | 2.29E-03 | 1.28E-08 | 33.55 |
| rs72768626 | 16 | G | A | -2.40E-02 | 4.09E-03 | 3.99E-09 | 36.59 |
| rs75120545 | 2 | T | C | -3.28E-02 | 5.67E-03 | 7.59E-09 | 21.96 |
| rs76640332 | 17 | A | G | -2.10E-02 | 2.39E-03 | 1.47E-18 | 73.82 |
| rs79616692 | 16 | C | G | 1.88E-02 | 3.15E-03 | 2.38E-09 | 36.68 |
| rs962961 | 14 | T | C | -1.22E-02 | 2.05E-03 | 2.78E-09 | 35.02 |

SNP: single nucleotide polymorphism; EA: Effect allele;OA:Other allele.

**Supplemental table 3: Predictive performance of lipidomic parameters using AUROC analysis.**

| **Variable Name** | **AUROC** | **Variable** |
| --- | --- | --- |
| 23568 | 0.66641 | Cholesterol in Medium HDL |
| 23569 | 0.665988 | Cholesteryl Esters in Medium HDL |
| 23566 | 0.665349 | Total Lipids in Medium HDL |
| 23565 | 0.6647 | Concentration of Medium HDL Particles |
| 23570 | 0.664011 | Free Cholesterol in Medium HDL |
| 23567 | 0.663934 | Phospholipids in Medium HDL |
| 23440 | 0.663206 | Apolipoprotein A1 |
| 23574 | 0.649549 | Phospholipids in Small HDL |
| 23575 | 0.644242 | Cholesterol in Small HDL |
| 23573 | 0.644038 | Total Lipids in Small HDL |
| 23576 | 0.641381 | Cholesteryl Esters in Small HDL |
| 23572 | 0.637033 | Concentration of Small HDL Particles |
| 23577 | 0.634277 | Free Cholesterol in Small HDL |
| 23437 | 0.63032 | Phosphatidylcholines |
| 23434 | 0.625841 | Phosphoglycerides |
| 23560 | 0.62523 | Phospholipids in Large HDL |
| 23436 | 0.622692 | Total Cholines |
| 23559 | 0.616955 | Total Lipids in Large HDL |
| 23455 | 0.61226 | Saturated Fatty Acids to Total Fatty Acids percentage |
| 23558 | 0.611683 | Concentration of Large HDL Particles |
| 23563 | 0.607983 | Free Cholesterol in Large HDL |
| 23561 | 0.605983 | Cholesterol in Large HDL |
| 23562 | 0.604522 | Cholesteryl Esters in Large HDL |
| 23438 | 0.598931 | Sphingomyelins |
| 23441 | 0.596871 | Apolipoprotein B to Apolipoprotein A1 ratio |
| 23433 | 0.591259 | Average Diameter for HDL Particles |
| 40062-2.0 | 0.589744 | cT1 |
| 23450 | 0.579997 | Docosahexaenoic Acid |
| 23456 | 0.573085 | Linoleic Acid to Total Fatty Acids percentage |
| 23448 | 0.569431 | Saturated Fatty Acids |
| 23527 | 0.565798 | Cholesteryl Esters in IDL |
| 23526 | 0.559897 | Cholesterol in IDL |
| 23499 | 0.556127 | Cholesteryl Esters in Large VLDL |
| 23476 | 0.555329 | Acetoacetate |
| 23477 | 0.55528 | Acetone |
| 23457 | 0.554952 | Docosahexaenoic Acid to Total Fatty Acids percentage |
| 23524 | 0.554687 | Total Lipids in IDL |
| 23498 | 0.552415 | Cholesterol in Large VLDL |
| AGE | 0.551576 | AGE |
| 23431 | 0.549459 | Average Diameter for VLDL Particles |
| 23555 | 0.548691 | Cholesteryl Esters in Very Large HDL |
| 23473 | 0.548384 | Citrate |
| 23500 | 0.548066 | Free Cholesterol in Large VLDL |
| 23432 | 0.54796 | Average Diameter for LDL Particles |
| 23444 | 0.54772 | Omega-3 Fatty Acids |
| 23492 | 0.546461 | Cholesteryl Esters in Very Large VLDL |
| 23442 | 0.545994 | Total Fatty Acids |
| 23474 | 0.545957 | 3-Hydroxybutyrate |
| 23495 | 0.54566 | Concentration of Large VLDL Particles |
| 23491 | 0.544951 | Cholesterol in Very Large VLDL |
| 23497 | 0.544485 | Phospholipids in Large VLDL |
| 23496 | 0.543923 | Total Lipids in Large VLDL |
| 23452 | 0.543277 | Omega-6 Fatty Acids to Total Fatty Acids percentage |
| 23509 | 0.543072 | Concentration of Small VLDL Particles |
| 23493 | 0.542182 | Free Cholesterol in Very Large VLDL |
| 23480 | 0.541943 | Glycoprotein Acetyls |
| 23479 | 0.541615 | Albumin |
| 23462 | 0.541318 | Glycine |
| 23528 | 0.541128 | Free Cholesterol in IDL |
| 23508 | 0.540576 | Triglycerides in Medium VLDL |
| 23461 | 0.540562 | Glutamine |
| 23515 | 0.540417 | Triglycerides in Small VLDL |
| 23535 | 0.540393 | Free Cholesterol in Large LDL |
| 23490 | 0.540152 | Phospholipids in Very Large VLDL |
| 23571 | 0.539551 | Triglycerides in Medium HDL |
| 23465 | 0.538997 | Isoleucine |
| 23446 | 0.538297 | Polyunsaturated Fatty Acids |
| 23501 | 0.538243 | Triglycerides in Large VLDL |
| 23532 | 0.538114 | Phospholipids in Large LDL |
| 23488 | 0.537889 | Concentration of Very Large VLDL Particles |
| 23525 | 0.537423 | Phospholipids in IDL |
| 23564 | 0.537268 | Triglycerides in Large HDL |
| 23454 | 0.537266 | Monounsaturated Fatty Acids to Total Fatty Acids percentage |
| 23551 | 0.536946 | Concentration of Very Large HDL Particles |
| 23489 | 0.536153 | Total Lipids in Very Large VLDL |
| 23531 | 0.535905 | Total Lipids in Large LDL |
| 23554 | 0.535722 | Cholesterol in Very Large HDL |
| 23552 | 0.534927 | Total Lipids in Very Large HDL |
| 23533 | 0.534689 | Cholesterol in Large LDL |
| 23553 | 0.534531 | Phospholipids in Very Large HDL |
| 23510 | 0.534476 | Total Lipids in Small VLDL |
| 23485 | 0.533661 | Cholesteryl Esters in Chylomicrons and Extremely Large VLDL |
| 23503 | 0.533333 | Total Lipids in Medium VLDL |
| 23453 | 0.533192 | Polyunsaturated Fatty Acids to Total Fatty Acids percentage |
| 23467 | 0.533139 | Valine |
| BMI | 0.532278 | Body mass index |
| 23534 | 0.532237 | Cholesteryl Esters in Large LDL |
| 23494 | 0.531391 | Triglycerides in Very Large VLDL |
| 23513 | 0.531207 | Cholesteryl Esters in Small VLDL |
| 23445 | 0.530989 | Omega-6 Fatty Acids |
| 23502 | 0.530987 | Concentration of Medium VLDL Particles |
| 23484 | 0.530265 | Cholesterol in Chylomicrons and Extremely Large VLDL |
| 23483 | 0.527638 | Phospholipids in Chylomicrons and Extremely Large VLDL |
| 23544 | 0.527255 | Concentration of Small LDL Particles |
| 23536 | 0.527098 | Triglycerides in Large LDL |
| 23512 | 0.526699 | Cholesterol in Small VLDL |
| 23451 | 0.526527 | Omega-3 Fatty Acids to Total Fatty Acids percentage |
| 23481 | 0.52592 | Concentration of Chylomicrons and Extremely Large VLDL Particles |
| 23504 | 0.525708 | Phospholipids in Medium VLDL |
| 23475 | 0.5255 | Acetate |
| 23511 | 0.52546 | Phospholipids in Small VLDL |
| 23486 | 0.525408 | Free Cholesterol in Chylomicrons and Extremely Large VLDL |
| PC5 | 0.524743 |  |
| 23507 | 0.524549 | Free Cholesterol in Medium VLDL |
| 23520 | 0.524472 | Cholesteryl Esters in Very Small VLDL |
| 23482 | 0.524188 | Total Lipids in Chylomicrons and Extremely Large VLDL |
| 23478 | 0.523981 | Creatinine |
| 23466 | 0.523951 | Leucine |
| SEX | 0.523624 |  |
| PC4 | 0.522139 |  |
| PC2 | 0.521939 |  |
| 23487 | 0.521865 | Triglycerides in Chylomicrons and Extremely Large VLDL |
| 23523 | 0.521362 | Concentration of IDL Particles |
| 23460 | 0.520503 | Alanine |
| 23529 | 0.519588 | Triglycerides in IDL |
| 23470 | 0.51958 | Glucose |
| PC9 | 0.519497 |  |
| 23542 | 0.519083 | Free Cholesterol in Medium LDL |
| 23472 | 0.518902 | Pyruvate |
| 23550 | 0.518606 | Triglycerides in Small LDL |
| 23514 | 0.518606 | Free Cholesterol in Small VLDL |
| 23447 | 0.518533 | Monounsaturated Fatty Acids |
| PC8 | 0.518402 |  |
| 23469 | 0.518189 | Tyrosine |
| 23519 | 0.517638 | Cholesterol in Very Small VLDL |
| 23505 | 0.514862 | Cholesterol in Medium VLDL |
| 23537 | 0.514223 | Concentration of Medium LDL Particles |
| 23539 | 0.513549 | Phospholipids in Medium LDL |
| 23538 | 0.512091 | Total Lipids in Medium LDL |
| 23540 | 0.511891 | Cholesterol in Medium LDL |
| PC1 | 0.511364 |  |
| 23546 | 0.510828 | Phospholipids in Small LDL |
| PC7 | 0.51078 |  |
| PC6 | 0.509444 |  |
| 23463 | 0.509355 | Histidine |
| 23578 | 0.508892 | Triglycerides in Small HDL |
| 23556 | 0.508678 | Free Cholesterol in Very Large HDL |
| 23522 | 0.50738 | Triglycerides in Very Small VLDL |
| 23517 | 0.50691 | Total Lipids in Very Small VLDL |
| 23557 | 0.5068 | Triglycerides in Very Large HDL |
| 23439 | 0.506712 | Apolipoprotein B |
| 23506 | 0.506096 | Cholesteryl Esters in Medium VLDL |
| 23471 | 0.505754 | Lactate |
| Ethnicity | 0.505724 |  |
| 23518 | 0.505201 | Phospholipids in Very Small VLDL |
| 23530 | 0.505126 | Concentration of Large LDL Particles |
| 23545 | 0.504657 | Total Lipids in Small LDL |
| 23543 | 0.503673 | Triglycerides in Medium LDL |
| 23548 | 0.501094 | Cholesteryl Esters in Small LDL |
| 23547 | 0.500328 | Cholesterol in Small LDL |
| 23468 | 0.500116 | Phenylalanine |
| PC10 | 0.500102 |  |
| 23521 | 0.499604 | Free Cholesterol in Very Small VLDL |
| 23516 | 0.499209 | Concentration of Very Small VLDL Particles |
| 23549 | 0.49888 | Free Cholesterol in Small LDL |
| 23449 | 0.498462 | Linoleic Acid |
| PC3 | 0.494293 |  |
| 23541 | 0.491426 | Cholesteryl Esters in Medium LDL |

**Supplemental table 4: Significant Enriched Pathways associated with differentially expressed metabolites in MetALD.**

| **Enriched Metabolite Sets** | **Total** | **Expected** | **Hits** | **Raw**  **p-value** | **Holm adjusted p-value** | **FDR adjusted p-value** |
| --- | --- | --- | --- | --- | --- | --- |
| Ketone Body Metabolism | 9 | 0.0126 | 3 | 1.28E-07 | 0.000425 | 0.000212 |
| Succinyl CoA: 3-ketoacid CoA transferase deficiency | 9 | 0.0126 | 3 | 1.28E-07 | 0.000425 | 0.000212 |
| Ketogenesis and ketolysis | 11 | 0.0154 | 3 | 2.51E-07 | 0.000833 | 0.000278 |
| Disorders in ketone body synthesis | 12 | 0.0168 | 3 | 3.35E-07 | 0.00111 | 0.000278 |
| Proton-coupled monocarboxylate transport | 7 | 0.00981 | 2 | 3.43E-05 | 0.114 | 0.0189 |
| Defective SLC16A1 causes symptomatic deficiency in lactate transport (SDLT) | 7 | 0.00981 | 2 | 3.43E-05 | 0.114 | 0.0189 |
| Disorders in ketolysis | 8 | 0.0112 | 2 | 4.57E-05 | 0.151 | 0.0216 |
| Utilization of Ketone Bodies | 9 | 0.0126 | 2 | 5.87E-05 | 0.194 | 0.0243 |
| Synthesis of Ketone Bodies | 13 | 0.0182 | 2 | 0.000127 | 0.42 | 0.0467 |
| Metabolism overview | 116 | 0.163 | 3 | 0.000365 | 1 | 0.121 |
| Fatty Acid Biosynthesis | 34 | 0.0477 | 2 | 9E-04 | 1 | 0.264 |
| Aspirin ADME | 35 | 0.0491 | 2 | 0.000954 | 1 | 0.264 |
| Leucine, isoleucine and valine metabolism | 67 | 0.0939 | 2 | 0.00348 | 1 | 0.887 |
| ApoE and miR-146 in inflammation and atherosclerosis | 3 | 0.0042 | 1 | 0.0042 | 1 | 0.985 |
| Transport of bile salts and organic acids, metal ions and amine compounds | 76 | 0.107 | 2 | 0.00446 | 1 | 0.985 |
| SLC transporter disorders | 80 | 0.112 | 2 | 0.00493 | 1 | 1 |
| Hydroxycarboxylic acid-binding receptors | 4 | 0.00561 | 1 | 0.0056 | 1 | 1 |
| SARS-CoV-2 and COVID-19 pathway | 5 | 0.00701 | 1 | 0.00699 | 1 | 1 |
| Disorders of transmembrane transporters | 98 | 0.137 | 2 | 0.00733 | 1 | 1 |
| Selenium micronutrient network | 106 | 0.149 | 2 | 0.00854 | 1 | 1 |
| Drug ADME | 110 | 0.154 | 2 | 0.00917 | 1 | 1 |
| Familial hyperlipidemia type 3 | 8 | 0.0112 | 1 | 0.0112 | 1 | 1 |
| Familial hyperlipidemia type 5 | 10 | 0.014 | 1 | 0.0139 | 1 | 1 |
| Familial  hyperlipidemia type 1 | 10 | 0.014 | 1 | 0.0139 | 1 | 1 |
| Lipid particles composition | 10 | 0.014 | 1 | 0.0139 | 1 | 1 |

**Supplemental table 5 : Association of genetically-predicted alcohol consumption with top 30 discriminatory metabolites in Mendelian randomization.**

| **Metabolites** | **Methods** | **N (SNP)** | **OR(95%CI)** | **P value** |
| --- | --- | --- | --- | --- |
| 3-Hydroxybutyrate | MR Egger | 32 | 1.71 (1.17,2.5) | 0.01 |
|  | Weighted median | 32 | 1.12 (0.95,1.31) | 0.194 |
|  | Inverse variance weighted | 32 | 1.04 (0.93,1.16) | 0.489 |
|  | Simple mode | 32 | 1.23 (0.83,1.81) | 0.319 |
|  | Weighted mode | 32 | 1.32 (0.93,1.86) | 0.129 |
| Acetoacetate | MR Egger | 33 | 2.15 (1.48,3.12) | <0.001 |
|  | Weighted median | 33 | 1.29 (1.1,1.52) | 0.002 |
|  | Inverse variance weighted | 33 | 1.23 (1.09,1.4) | 0.001 |
|  | Simple mode | 33 | 1.23 (0.82,1.84) | 0.322 |
|  | Weighted mode | 33 | 1.5 (1.17,1.94) | 0.004 |
| Concentration of Large HDL Particles | MR Egger | 31 | 0.92 (0.62,1.38) | 0.693 |
|  | Weighted median | 31 | 1.11 (0.94,1.31) | 0.222 |
|  | Inverse variance weighted | 31 | 1.2 (1.06,1.36) | 0.005 |
|  | Simple mode | 31 | 0.92 (0.65,1.31) | 0.65 |
|  | Weighted mode | 31 | 1.01 (0.79,1.28) | 0.94 |
| Cholesteryl Esters in Large HDL | MR Egger | 31 | 0.88 (0.59,1.31) | 0.525 |
|  | Weighted median | 31 | 1.01 (0.86,1.18) | 0.918 |
|  | Inverse variance weighted | 31 | 1.14 (1.01,1.3) | 0.041 |
|  | Simple mode | 31 | 0.91 (0.62,1.32) | 0.611 |
|  | Weighted mode | 31 | 0.94 (0.76,1.16) | 0.563 |
| Cholesterol in Large HDL | MR Egger | 31 | 0.88 (0.59,1.32) | 0.548 |
|  | Weighted median | 31 | 1.01 (0.86,1.18) | 0.915 |
|  | Inverse variance weighted | 31 | 1.15 (1.01,1.3) | 0.03 |
|  | Simple mode | 31 | 0.91 (0.63,1.3) | 0.596 |
|  | Weighted mode | 31 | 0.94 (0.76,1.16) | 0.565 |
| Free Cholesterol in Large HDL | MR Egger | 31 | 0.9 (0.6,1.34) | 0.604 |
|  | Weighted median | 31 | 1.03 (0.88,1.2) | 0.75 |
|  | Inverse variance weighted | 31 | 1.17 (1.03,1.33) | 0.015 |
|  | Simple mode | 31 | 0.92 (0.66,1.28) | 0.615 |
|  | Weighted mode | 31 | 0.96 (0.77,1.19) | 0.712 |
| Phospholipids in Large HDL | MR Egger | 31 | 0.98 (0.7,1.38) | 0.915 |
|  | Weighted median | 31 | 1.14 (0.97,1.33) | 0.102 |
|  | Inverse variance weighted | 31 | 1.23 (1.11,1.37) | <0.001 |
|  | Simple mode | 31 | 1.03 (0.74,1.42) | 0.866 |
|  | Weighted mode | 31 | 1.07 (0.86,1.35) | 0.543 |
| Total Lipids in Large HDL | MR Egger | 31 | 0.93 (0.64,1.34) | 0.7 |
|  | Weighted median | 31 | 1.1 (0.93,1.28) | 0.261 |
|  | Inverse variance weighted | 31 | 1.2 (1.07,1.35) | 0.002 |
|  | Simple mode | 31 | 0.94 (0.67,1.31) | 0.708 |
|  | Weighted mode | 31 | 1 (0.8,1.26) | 0.99 |
| Acetone | MR Egger | 33 | 1.39 (0.89,2.18) | 0.154 |
|  | Weighted median | 33 | 1.03 (0.86,1.22) | 0.766 |
|  | Inverse variance weighted | 33 | 1.12 (0.98,1.28) | 0.102 |
|  | Simple mode | 33 | 0.9 (0.59,1.36) | 0.606 |
|  | Weighted mode | 33 | 0.92 (0.65,1.31) | 0.66 |
| Free Cholesterol in Medium HDL | MR Egger | 32 | 1.58 (1.09,2.3) | 0.022 |
|  | Weighted median | 32 | 1.59 (1.35,1.87) | <0.001 |
|  | Inverse variance weighted | 32 | 1.56 (1.39,1.75) | <0.001 |
|  | Simple mode | 32 | 1.44 (1.01,2.05) | 0.052 |
|  | Weighted mode | 32 | 1.56 (1.18,2.05) | 0.003 |
| Cholesterol in Medium HDL | MR Egger | 32 | 1.56 (1.1,2.23) | 0.018 |
|  | Weighted median | 32 | 1.57 (1.33,1.84) | <0.001 |
|  | Inverse variance weighted | 32 | 1.54 (1.38,1.71) | <0.001 |
|  | Simple mode | 32 | 1.46 (1.02,2.09) | 0.048 |
|  | Weighted mode | 32 | 1.58 (1.21,2.06) | 0.002 |
| Cholesteryl Esters in Medium HDL | MR Egger | 32 | 1.55 (1.09,2.21) | 0.02 |
|  | Weighted median | 32 | 1.56 (1.32,1.84) | <0.001 |
|  | Inverse variance weighted | 32 | 1.53 (1.37,1.7) | <0.001 |
|  | Simple mode | 32 | 1.39 (0.96,2) | 0.088 |
|  | Weighted mode | 32 | 1.54 (1.18,2.02) | 0.003 |
| Concentration of Medium HDL Particles | MR Egger | 31 | 1.93 (1.37,2.72) | 0.001 |
|  | Weighted median | 31 | 1.73 (1.48,2.02) | <0.001 |
|  | Inverse variance weighted | 31 | 1.66 (1.49,1.84) | <0.001 |
|  | Simple mode | 31 | 1.75 (1.27,2.41) | 0.002 |
|  | Weighted mode | 31 | 1.77 (1.4,2.25) | <0.001 |
| Total Lipids in Medium HDL | MR Egger | 31 | 2.03 (1.44,2.86) | <0.001 |
|  | Weighted median | 31 | 1.81 (1.54,2.12) | <0.001 |
|  | Inverse variance weighted | 31 | 1.69 (1.52,1.87) | <0.001 |
|  | Simple mode | 31 | 1.81 (1.32,2.5) | 0.001 |
|  | Weighted mode | 31 | 1.83 (1.44,2.32) | <0.001 |
| Phospholipids in Medium HDL | MR Egger | 31 | 2.15 (1.51,3.06) | <0.001 |
|  | Weighted median | 31 | 1.86 (1.58,2.18) | <0.001 |
|  | Inverse variance weighted | 31 | 1.72 (1.54,1.92) | <0.001 |
|  | Simple mode | 31 | 1.84 (1.31,2.6) | 0.002 |
|  | Weighted mode | 31 | 1.88 (1.48,2.39) | <0.001 |
| Docosahexaenoic Acid | MR Egger | 31 | 1.29 (0.78,2.15) | 0.333 |
|  | Weighted median | 31 | 1.2 (1.01,1.43) | 0.037 |
|  | Inverse variance weighted | 31 | 1.29 (1.11,1.49) | 0.001 |
|  | Simple mode | 31 | 1.11 (0.75,1.63) | 0.612 |
|  | Weighted mode | 31 | 1.18 (0.91,1.54) | 0.229 |
| Apolipoprotein A1 | MR Egger | 31 | 1.86 (1.32,2.61) | 0.001 |
|  | Weighted median | 31 | 1.69 (1.44,1.98) | <0.001 |
|  | Inverse variance weighted | 31 | 1.64 (1.48,1.82) | <0.001 |
|  | Simple mode | 31 | 1.43 (1.02,2.01) | 0.046 |
|  | Weighted mode | 31 | 1.65 (1.27,2.13) | 0.001 |
| Phosphatidylcholines | MR Egger | 29 | 1.82 (1.22,2.72) | 0.007 |
|  | Weighted median | 29 | 1.63 (1.37,1.94) | <0.001 |
|  | Inverse variance weighted | 29 | 1.56 (1.38,1.77) | <0.001 |
|  | Simple mode | 29 | 1.64 (1.08,2.48) | 0.028 |
|  | Weighted mode | 29 | 1.83 (1.35,2.49) | 0.001 |
| Phospholipids in Small HDL | MR Egger | 32 | 2.18 (1.39,3.41) | 0.002 |
|  | Weighted median | 32 | 1.73 (1.44,2.08) | <0.001 |
|  | Inverse variance weighted | 32 | 1.67 (1.45,1.92) | <0.001 |
|  | Simple mode | 32 | 2.13 (1.34,3.38) | 0.003 |
|  | Weighted mode | 32 | 2.22 (1.23,4.03) | 0.013 |
| Phosphoglycerides | MR Egger | 29 | 1.94 (1.33,2.85) | 0.002 |
|  | Weighted median | 29 | 1.62 (1.36,1.93) | <0.001 |
|  | Inverse variance weighted | 29 | 1.59 (1.41,1.8) | <0.001 |
|  | Simple mode | 29 | 1.47 (1.03,2.09) | 0.043 |
|  | Weighted mode | 29 | 1.84 (1.36,2.47) | <0.001 |
| Cholesteryl Esters in Small HDL | MR Egger | 33 | 1.89 (1.22,2.94) | 0.008 |
|  | Weighted median | 33 | 1.53 (1.29,1.82) | <0.001 |
|  | Inverse variance weighted | 33 | 1.54 (1.35,1.77) | <0.001 |
|  | Simple mode | 33 | 1.52 (1.06,2.17) | 0.03 |
|  | Weighted mode | 33 | 1.48 (0.98,2.22) | 0.07 |
| Cholesterol in Small HDL | MR Egger | 32 | 1.95 (1.31,2.9) | 0.002 |
|  | Weighted median | 32 | 1.6 (1.33,1.92) | <0.001 |
|  | Inverse variance weighted | 32 | 1.56 (1.38,1.76) | <0.001 |
|  | Simple mode | 32 | 1.68 (1.14,2.48) | 0.014 |
|  | Weighted mode | 32 | 1.78 (1.08,2.94) | 0.032 |
| Total Lipids in Small HDL | MR Egger | 32 | 2.18 (1.39,3.41) | 0.002 |
|  | Weighted median | 32 | 1.75 (1.46,2.11) | <0.001 |
|  | Inverse variance weighted | 32 | 1.64 (1.42,1.88) | <0.001 |
|  | Simple mode | 32 | 2.11 (1.3,3.4) | 0.005 |
|  | Weighted mode | 32 | 1.13 (0.56,2.27) | 0.741 |
| Free Cholesterol in Small HDL | MR Egger | 31 | 1.86 (1.17,2.98) | 0.014 |
|  | Weighted median | 31 | 1.57 (1.31,1.88) | <0.001 |
|  | Inverse variance weighted | 31 | 1.69 (1.46,1.95) | <0.001 |
|  | Simple mode | 31 | 1.32 (0.89,1.98) | 0.181 |
|  | Weighted mode | 31 | 1.3 (0.73,2.3) | 0.38 |
| Total Cholines | MR Egger | 30 | 1.51 (1.02,2.25) | 0.051 |
|  | Weighted median | 30 | 1.59 (1.34,1.88) | <0.001 |
|  | Inverse variance weighted | 30 | 1.47 (1.3,1.67) | <0.001 |
|  | Simple mode | 30 | 1.73 (1.15,2.61) | 0.014 |
|  | Weighted mode | 30 | 1.82 (1.29,2.56) | 0.002 |
| Concentration of Small HDL Particles | MR Egger | 33 | 1.96 (1.24,3.09) | 0.007 |
|  | Weighted median | 33 | 1.67 (1.41,1.97) | <0.001 |
|  | Inverse variance weighted | 33 | 1.6 (1.39,1.84) | <0.001 |
|  | Simple mode | 33 | 1.72 (1.15,2.57) | 0.013 |
|  | Weighted mode | 33 | 1.84 (1.07,3.17) | 0.034 |
| met-d-Sphingomyelins | MR Egger | 30 | 1.4 (0.96,2.04) | 0.095 |
|  | Weighted median | 30 | 1.32 (1.13,1.55) | <0.001 |
|  | Inverse variance weighted | 30 | 1.27 (1.13,1.44) | <0.001 |
|  | Simple mode | 30 | 1.35 (0.97,1.87) | 0.083 |
|  | Weighted mode | 30 | 1.38 (1.08,1.78) | 0.017 |
| Average Diameter for HDL Particles | MR Egger | 31 | 0.79 (0.54,1.14) | 0.219 |
|  | Weighted median | 31 | 1.02 (0.87,1.2) | 0.818 |
|  | Inverse variance weighted | 31 | 1.11 (0.98,1.25) | 0.103 |
|  | Simple mode | 31 | 0.93 (0.65,1.33) | 0.707 |
|  | Weighted mode | 31 | 0.95 (0.76,1.18) | 0.649 |
| Linoleic Acid to Total Fatty Acids percentage | MR Egger | 32 | 0.68 (0.42,1.1) | 0.13 |
|  | Weighted median | 32 | 0.77 (0.65,0.92) | 0.004 |
|  | Inverse variance weighted | 32 | 0.75 (0.64,0.87) | <0.001 |
|  | Simple mode | 32 | 0.84 (0.57,1.24) | 0.387 |
|  | Weighted mode | 32 | 0.79 (0.62,1.01) | 0.066 |
| Apolipoprotein B to Apolipoprotein A1 ratio | MR Egger | 31 | 0.89 (0.56,1.41) | 0.622 |
|  | Weighted median | 31 | 0.81 (0.67,0.97) | 0.024 |
|  | Inverse variance weighted | 31 | 0.74 (0.64,0.86) | <0.001 |
|  | Simple mode | 31 | 0.68 (0.45,1.02) | 0.075 |
|  | Weighted mode | 31 | 0.81 (0.62,1.06) | 0.132 |

HDL: High density lipoprotein.

**Supplemental table 6: Heterogeneity analysis results of mendelian randomization analysis results between alcohol consumption and top 30 discriminatory metabolites.**

| **Metabolite** | **Method** | **Q** | **Q_df** | **Q_pval** |
| --- | --- | --- | --- | --- |
| 3-Hydroxybutyrate levels | MR Egger | 24.83 | 30 | 0.733 |
|  | IVW | 31.91 | 31 | 0.421 |
| Acetoacetate levels | MR Egger | 35.21 | 31 | 0.276 |
|  | IVW | 45.99 | 32 | 0.052 |
| Concentration of large HDL particles | MR Egger | 46.39 | 29 | 0.021 |
|  | IVW | 49.30 | 30 | 0.015 |
| Cholesteryl esters in large HDL | MR Egger | 46.04 | 29 | 0.023 |
|  | IVW | 48.94 | 30 | 0.016 |
| Cholesterol in large HDL | MR Egger | 45.62 | 29 | 0.026 |
|  | IVW | 48.52 | 30 | 0.018 |
| Free cholesterol in large HDL | MR Egger | 46.22 | 29 | 0.022 |
|  | IVW | 49.15 | 30 | 0.015 |
| Phospholipids in large HDL | MR Egger | 32.91 | 29 | 0.282 |
|  | IVW | 35.07 | 30 | 0.240 |
| Total lipids in large HDL | MR Egger | 39.29 | 29 | 0.096 |
|  | IVW | 42.05 | 30 | 0.071 |
| Acetone levels | MR Egger | 50.55 | 31 | 0.015 |
|  | IVW | 52.21 | 32 | 0.013 |
| Free cholesterol in medium HDL | MR Egger | 41.01 | 30 | 0.087 |
|  | IVW | 41.02 | 31 | 0.108 |
| Cholesterol in medium HDL | MR Egger | 35.60 | 30 | 0.222 |
|  | IVW | 35.61 | 31 | 0.260 |
| Cholesteryl esters in medium HDL | MR Egger | 35.49 | 30 | 0.225 |
|  | IVW | 35.50 | 31 | 0.264 |
| Concentration of medium HDL particles | MR Egger | 26.27 | 29 | 0.611 |
|  | IVW | 27.12 | 30 | 0.617 |
| Total lipids in medium HDL | MR Egger | 28.02 | 29 | 0.517 |
|  | IVW | 29.23 | 30 | 0.506 |
| Phospholipids in medium HDL | MR Egger | 29.84 | 29 | 0.422 |
|  | IVW | 31.58 | 30 | 0.387 |
| Docosahexaenoic acid levels | MR Egger | 56.23 | 29 | 0.002 |
|  | IVW | 56.23 | 30 | 0.003 |
| Apolipoprotein A1 levels | MR Egger | 29.28 | 29 | 0.451 |
|  | IVW | 29.85 | 30 | 0.473 |
| Phosphatidylcholines | MR Egger | 35.72 | 27 | 0.121 |
|  | IVW | 36.52 | 28 | 0.130 |
| Phospholipids in small HDL | MR Egger | 49.47 | 30 | 0.014 |
|  | IVW | 51.91 | 31 | 0.011 |
| Phosphoglycerides levels | MR Egger | 31.67 | 27 | 0.245 |
|  | IVW | 33.03 | 28 | 0.235 |
| Cholesteryl esters in small HDL | MR Egger | 48.62 | 31 | 0.023 |
|  | IVW | 50.03 | 32 | 0.022 |
| Cholesterol in small HDL | MR Egger | 38.46 | 30 | 0.138 |
|  | IVW | 40.23 | 31 | 0.124 |
| Total lipids in small HDL | MR Egger | 48.80 | 30 | 0.016 |
|  | IVW | 51.60 | 31 | 0.012 |
| Free cholesterol in small HDL | MR Egger | 53.16 | 29 | 0.004 |
|  | IVW | 53.52 | 30 | 0.005 |
| Total cholines levels | MR Egger | 40.46 | 28 | 0.060 |
|  | IVW | 40.49 | 29 | 0.076 |
| Concentration of small HDL particles | MR Egger | 51.92 | 31 | 0.011 |
|  | IVW | 53.32 | 32 | 0.010 |
| Sphingomyelins | MR Egger | 37.14 | 28 | 0.116 |
|  | IVW | 37.48 | 29 | 0.134 |
| Average diameter for HDL particles | MR Egger | 39.56 | 29 | 0.091 |
|  | IVW | 44.36 | 30 | 0.044 |
| Ratio of linoleic acid to total fatty acids | MR Egger | 61.43 | 30 | 0.001 |
|  | IVW | 61.75 | 31 | 0.001 |
| Ratio of apolipoprotein B to apolipoprotein A1 levels | MR Egger | 48.15 | 29 | 0.014 |
|  | IVW | 49.24 | 30 | 0.015 |

HDL: High density lipoprotein; IVW: Inverse variance weighted

**Supplemental table 7: MR-Egger regression intercept analysis for pleiotropy testing in the association between alcohol consumption and top 30 discriminatory metabolites.**

| **Metabolite** | **Egger_intercept** | **Se_value** | **P_value** |
| --- | --- | --- | --- |
| 3-Hydroxybutyrate levels | -7.71E-03 | 2.90E-03 | 0.012 |
| Acetoacetate levels | -8.83E-03 | 2.87E-03 | 0.004 |
| Concentration of large HDL particles | 4.12E-03 | 3.06E-03 | 0.188 |
| Cholesteryl esters in large HDL | 4.15E-03 | 3.07E-03 | 0.187 |
| Cholesterol in large HDL | 4.14E-03 | 3.05E-03 | 0.185 |
| Free cholesterol in large HDL | 4.14E-03 | 3.05E-03 | 0.185 |
| Phospholipids in large HDL | 3.57E-03 | 2.59E-03 | 0.178 |
| Total lipids in large HDL | 4.03E-03 | 2.82E-03 | 0.164 |
| Acetone levels | -3.48E-03 | 3.45E-03 | 0.321 |
| Free cholesterol in medium HDL | -2.52E-04 | 2.89E-03 | 0.931 |
| Cholesterol in medium HDL | -2.77E-04 | 2.72E-03 | 0.919 |
| Cholesteryl esters in medium HDL | -2.59E-04 | 2.73E-03 | 0.925 |
| Concentration of medium HDL particles | -2.38E-03 | 2.59E-03 | 0.366 |
| Total lipids in medium HDL | -2.88E-03 | 2.61E-03 | 0.280 |
| Phospholipids in medium HDL | -3.47E-03 | 2.67E-03 | 0.204 |
| Docosahexaenoic acid levels | -6.56E-05 | 3.80E-03 | 0.986 |
| Apolipoprotein A1 levels | -1.95E-03 | 2.59E-03 | 0.456 |
| Phosphatidylcholines | -2.37E-03 | 3.05E-03 | 0.445 |
| Phospholipids in small HDL | -4.24E-03 | 3.48E-03 | 0.233 |
| Phosphoglycerides levels | -3.12E-03 | 2.90E-03 | 0.291 |
| Cholesteryl esters in small HDL | -3.23E-03 | 3.40E-03 | 0.350 |
| Cholesterol in small HDL | -3.60E-03 | 3.07E-03 | 0.250 |
| Total lipids in small HDL | -4.56E-03 | 3.48E-03 | 0.200 |
| Free cholesterol in small HDL | -1.60E-03 | 3.63E-03 | 0.664 |
| Total cholines levels | -4.41E-04 | 3.09E-03 | 0.887 |
| Concentration of small HDL particles | -3.22E-03 | 3.51E-03 | 0.367 |
| Sphingomyelins | -1.50E-03 | 2.95E-03 | 0.616 |
| Average diameter for HDL particles | 5.35E-03 | 2.85E-03 | 0.071 |
| Ratio of linoleic acid to total fatty acids | 1.48E-03 | 3.74E-03 | 0.695 |
| Ratio of apolipoprotein B to apolipoprotein A1 levels | -2.83E-03 | 3.50E-03 | 0.425 |

HDL: High density lipoprotein;

**Supplemental table 8: MR-PRESSO analysis results for detecting outliers in the association between alcohol consumption and top 30 discriminatory metabolites.**

| **Metabolites** | **Raw MR results** | | | **Corrected MR results** | | | |
| --- | --- | --- | --- | --- | --- | --- | --- |
|  | **Causal Estimate** | **Sd value** | **P**  **value** | **Causal Estimate** | **Sd value** | **OR (95%VI)** | **P**  **value** |
| 3-Hydroxybutyrate levels | 0.14 | 0.12 | 0.227 | 0.04 | 0.06 | 1.04 (0.93,1.16) | 0.494 |
| Acetoacetate levels | 0.21 | 0.07 | 0.003 | NA | NA | NA | NA |
| Concentration of large HDL particles | 0.29 | 0.09 | 0.004 | 0.18 | 0.06 | 1.2 (1.06,1.36) | 0.009 |
| Cholesteryl esters in large HDL | 0.24 | 0.09 | 0.014 | 0.13 | 0.06 | 1.14 (1.01,1.3) | 0.050 |
| Cholesterol in large HDL | 0.25 | 0.09 | 0.011 | 0.14 | 0.06 | 1.15 (1.01,1.3) | 0.038 |
| Free cholesterol in large HDL | 0.27 | 0.09 | 0.007 | 0.16 | 0.06 | 1.17 (1.03,1.33) | 0.021 |
| Phospholipids in large HDL | 0.32 | 0.09 | 0.001 | 0.21 | 0.05 | 1.23 (1.11,1.37) | 0.001 |
| Total lipids in large HDL | 0.3 | 0.09 | 0.003 | 0.18 | 0.06 | 1.2 (1.07,1.35) | 0.005 |
| Acetone levels | 0.11 | 0.07 | 0.112 | NA | NA | NA | NA |
| Free cholesterol in medium HDL | 0.51 | 0.09 | <0.001 | 0.44 | 0.06 | 1.56 (1.39,1.75) | <0.001 |
| Cholesterol in medium HDL | 0.5 | 0.08 | <0.001 | 0.43 | 0.05 | 1.54 (1.38,1.71) | <0.001 |
| Cholesteryl esters in medium HDL | 0.49 | 0.08 | <0.001 | 0.42 | 0.06 | 1.53 (1.37,1.7) | <0.001 |
| Concentration of medium HDL particles | 0.53 | 0.08 | <0.001 | 0.51 | 0.05 | 1.66 (1.5,1.83) | <0.001 |
| Total lipids in medium HDL | 0.54 | 0.08 | <0.001 | 0.52 | 0.05 | 1.69 (1.52,1.87) | <0.001 |
| Phospholipids in medium HDL | 0.56 | 0.08 | <0.001 | 0.54 | 0.06 | 1.72 (1.54,1.92) | <0.001 |
| Docosahexaenoic acid levels | 0.29 | 0.1 | 0.005 | 0.25 | 0.08 | 1.29 (1.11,1.49) | 0.002 |
| Apolipoprotein A1 levels | 0.52 | 0.08 | <0.001 | 0.49 | 0.05 | 1.64 (1.48,1.82) | <0.001 |
| Phosphatidylcholines | 0.48 | 0.1 | <0.001 | 0.45 | 0.06 | 1.56 (1.38,1.77) | <0.001 |
| Phospholipids in small HDL | 0.54 | 0.08 | <0.001 | 0.51 | 0.07 | 1.67 (1.45,1.92) | <0.001 |
| Phosphoglycerides levels | 0.49 | 0.1 | <0.001 | 0.47 | 0.06 | 1.59 (1.41,1.8) | <0.001 |
| Cholesteryl esters in small HDL | 0.43 | 0.07 | <0.001 | NA | NA | NA | NA |
| Cholesterol in small HDL | 0.48 | 0.07 | <0.001 | 0.44 | 0.06 | 1.56 (1.38,1.76) | <0.001 |
| Total lipids in small HDL | 0.52 | 0.08 | <0.001 | 0.49 | 0.07 | 1.64 (1.42,1.88) | <0.001 |
| Free cholesterol in small HDL | 0.54 | 0.09 | <0.001 | 0.52 | 0.07 | 1.69 (1.46,1.95) | <0.001 |
| Total cholines levels | 0.46 | 0.1 | <0.001 | 0.39 | 0.06 | 1.47 (1.3,1.67) | <0.001 |
| Concentration of small HDL particles | 0.47 | 0.07 | <0.001 | NA | NA | NA | NA |
| Sphingomyelins | 0.32 | 0.1 | 0.003 | 0.24 | 0.06 | 1.27 (1.13,1.44) | <0.001 |
| Average diameter for HDL particles | 0.21 | 0.09 | 0.023 | 0.1 | 0.06 | 1.11 (0.98,1.25) | 0.113 |
| Ratio of linoleic acid to total fatty acids | -0.32 | 0.08 | <0.001 | -0.29 | 0.08 | 0.75 (0.64,0.87) | 0.001 |
| Ratio of apolipoprotein B to apolipoprotein A1 levels | -0.28 | 0.09 | 0.003 | -0.3 | 0.07 | 0.74 (0.64,0.86) | <0.001 |

HDL: High density lipoprotein;

**
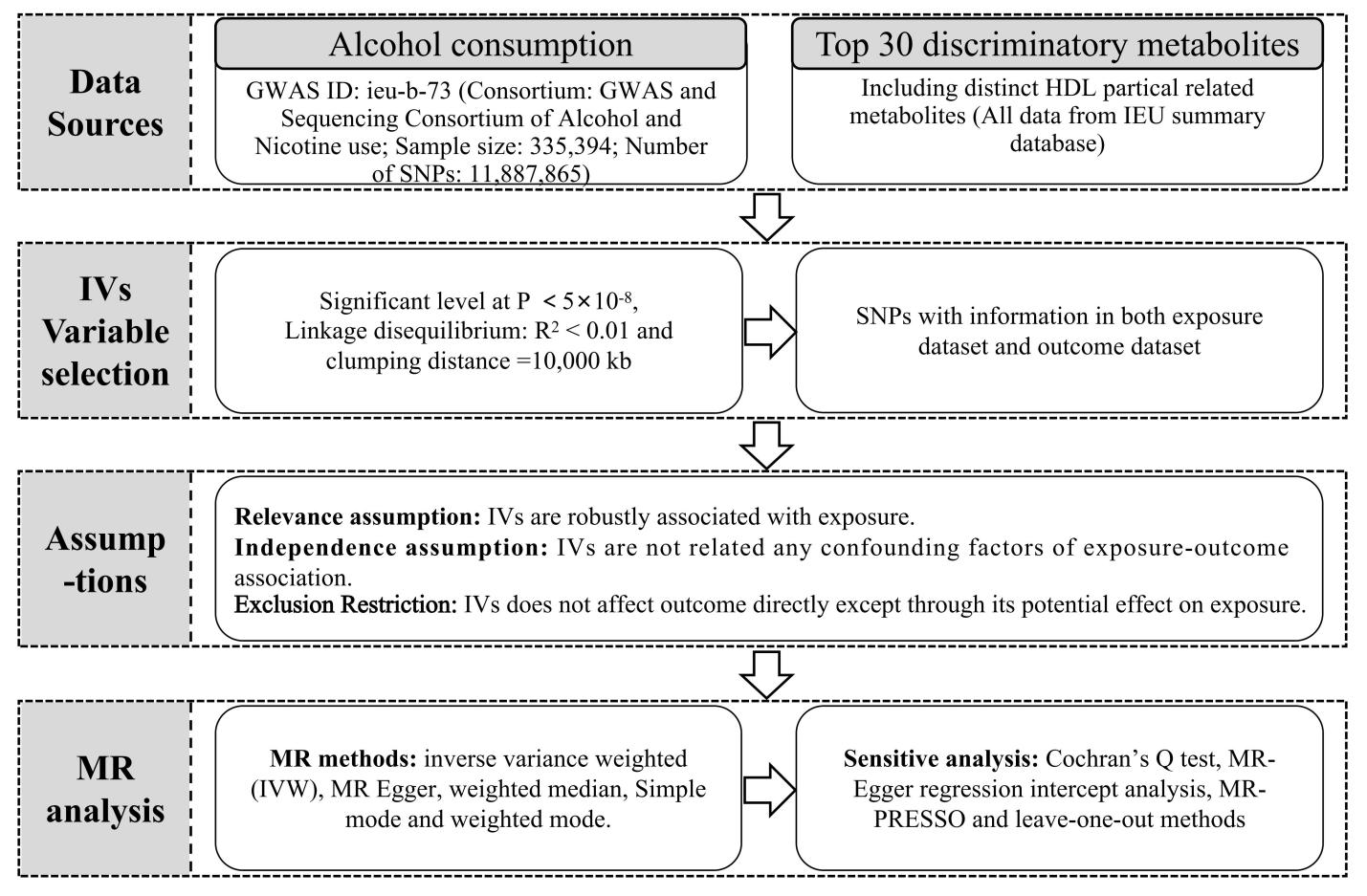
**

**Supplemental Figure 1: The flowchart and three key assumption of mendelian randomization.**

**Supplemental Figure 2: Cubic spline modelling the relationship between baseline HDL and MetALD risk compared to MASLD**

**Supplemental Figure 3: Cubic spline modelling the relationship between Acetoacetate and MetALD risk compared to MASLD**

**
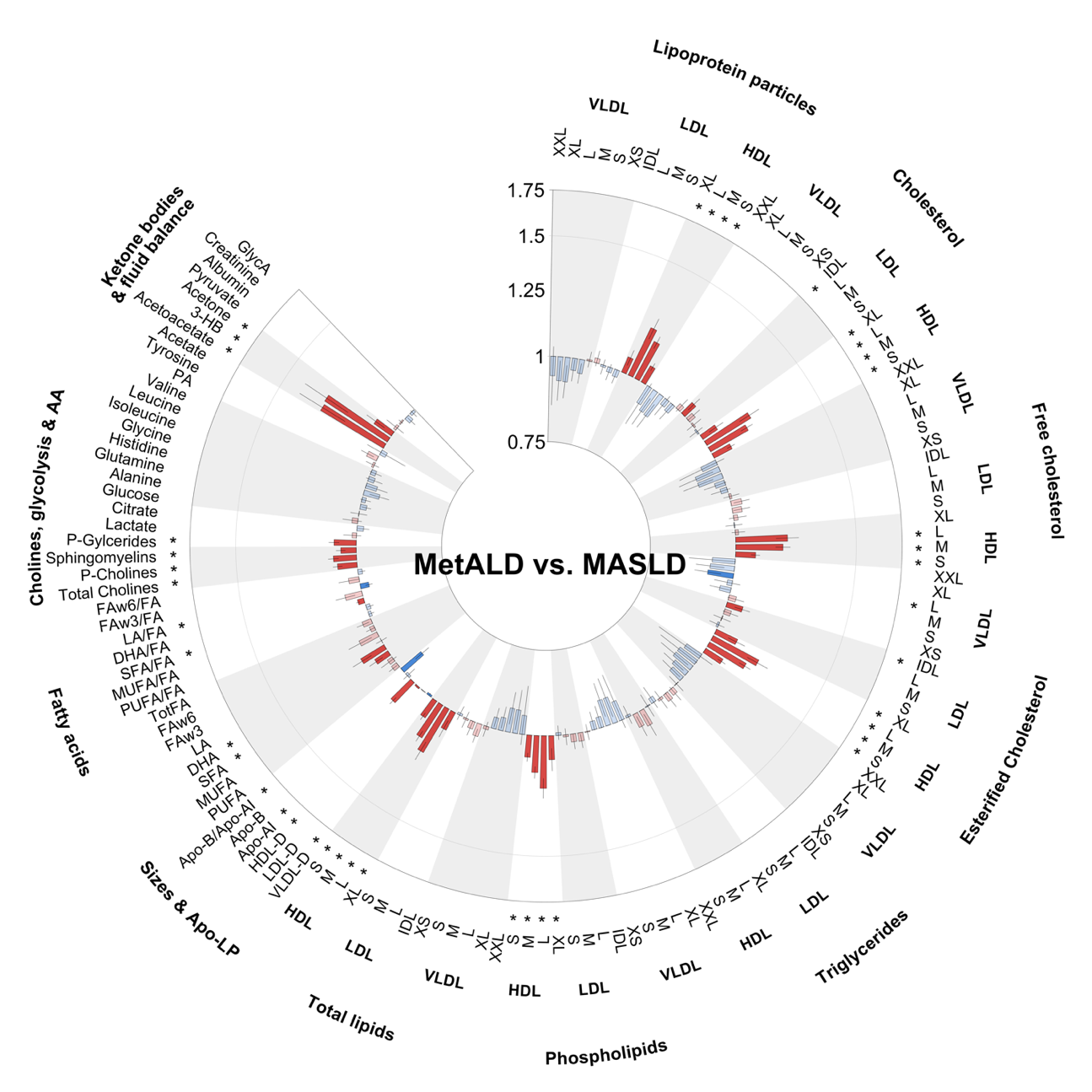
**

**Supplemental Figure 4: Circle plot for lipidomic analysis for MetALD vs MASLD UKB participants at baseline corrected for cT1.** Red=Higher in MetALD. blue = higher in MASLD. Lipidomic parameters were measured through NMR spectroscopy. Hazard ratios (with 95% confidence intervals) are presented per 1-SD higher metabolic biomarker on the natural log scale. stratified by age. sex. body mass index. and Townsend deprivation index. * The Bonferroni method was employed to correct for multiple comparisons (p<0.05/249). Original code by Diego J Aguilar-Ramirez. Abbreviations: Apo-A1 - Apolipoprotein A1. Apo-B - Apolipoprotein B. FA - Fatty Acids. FAw3 - Omega-3 Fatty Acids. FAw6 - Omega-6 Fatty Acids. HDL - High-Density Lipoprotein. IDL - Intermediate-Density Lipoprotein. LA - Linoleic Acid. LDL - Low-Density Lipoprotein. M - Medium (lipid particle size). MUFA - Monounsaturated Fatty Acids. PUFA - Polyunsaturated Fatty Acids. S - Small (lipid particle size). SFA - Saturated Fatty Acids. TG - Triglycerides. VLDL - Very Low-Density Lipoprotein. XL - Extra Large (lipid particle size). XXL - Extra Extra Large (lipid particle size). 3-HB - 3-Hydroxybutyrate. P-Glycerides - Phosphoglycerides. P-Cholines - Phosphatidylcholines. DHA - Docosahexaenoic Acid.

**Supplemental Figure 5: Correlation of Acetoacetate at baseline and follow up (R2=0.11)**

**Supplemental Figure 6: Correlation of Phospholipids in Large HDL at baseline and follow up (R2=0.83)**


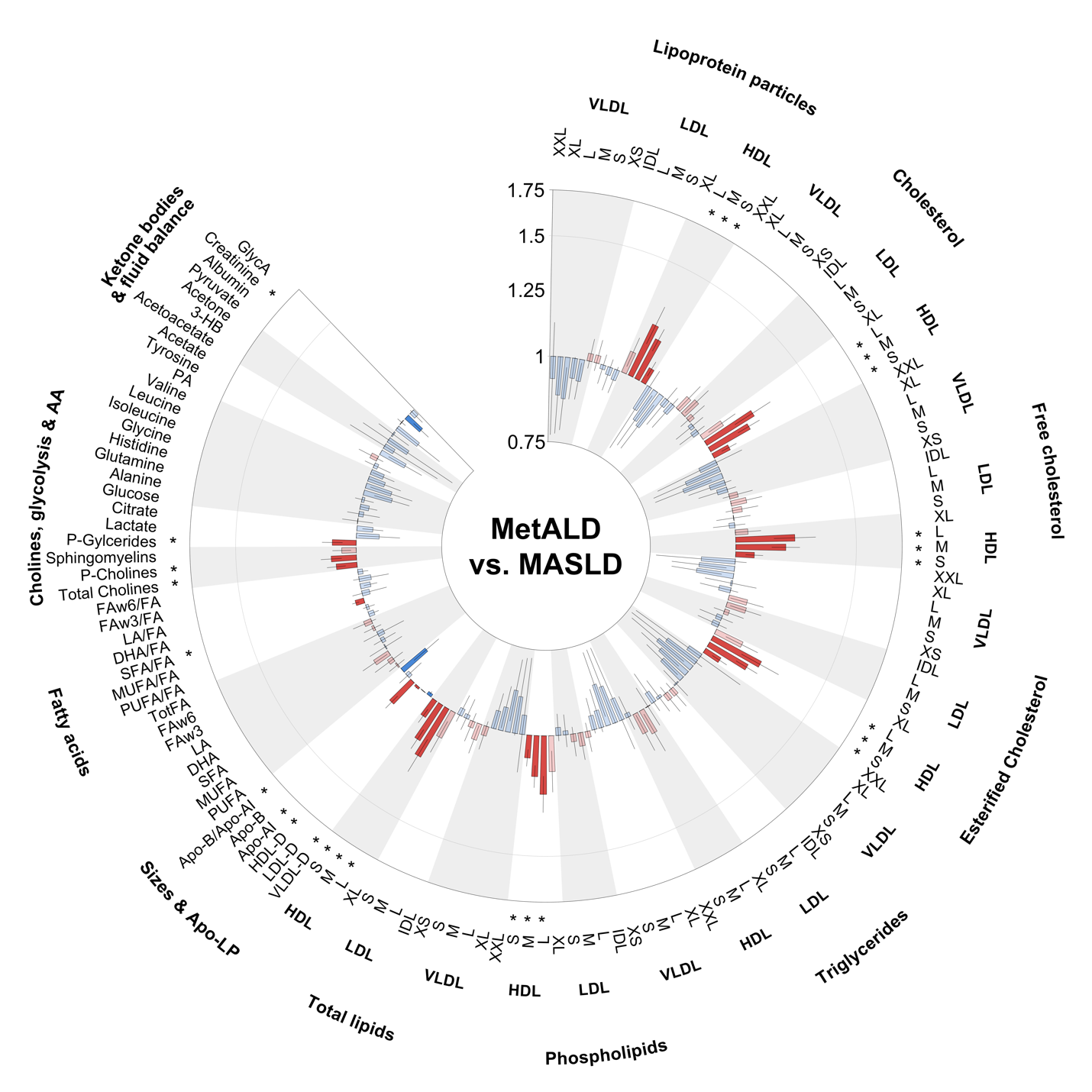


**Supplemental Figure 7: Circle plot for lipidomic analysis for MetALD vs MASLD UKB participants at follow up.** Red=Higher in MetALD. blue = higher in MASLD. Lipidomic parameters were measured through NMR spectroscopy. Hazard ratios (with 95% confidence intervals) are presented per 1-SD higher metabolic biomarker on the natural log scale. stratified by age. sex. body mass index. and Townsend deprivation index. * The Bonferroni method was employed to correct for multiple comparisons (p<0.05/249). Original code by Diego J Aguilar-Ramirez. Abbreviations: Apo-A1 - Apolipoprotein A1. Apo-B - Apolipoprotein B. FA - Fatty Acids. FAw3 - Omega-3 Fatty Acids. FAw6 - Omega-6 Fatty Acids. HDL - High-Density Lipoprotein. IDL - Intermediate-Density Lipoprotein. LA - Linoleic Acid. LDL - Low-Density Lipoprotein. M - Medium (lipid particle size). MUFA - Monounsaturated Fatty Acids. PUFA - Polyunsaturated Fatty Acids. S - Small (lipid particle size). SFA - Saturated Fatty Acids. TG - Triglycerides. VLDL - Very Low-Density Lipoprotein. XL - Extra Large (lipid particle size). XXL - Extra Extra Large (lipid particle size). 3-HB - 3-Hydroxybutyrate. P-Glycerides - Phosphoglycerides. P-Cholines - Phosphatidylcholines. DHA - Docosahexaenoic Acid.

**
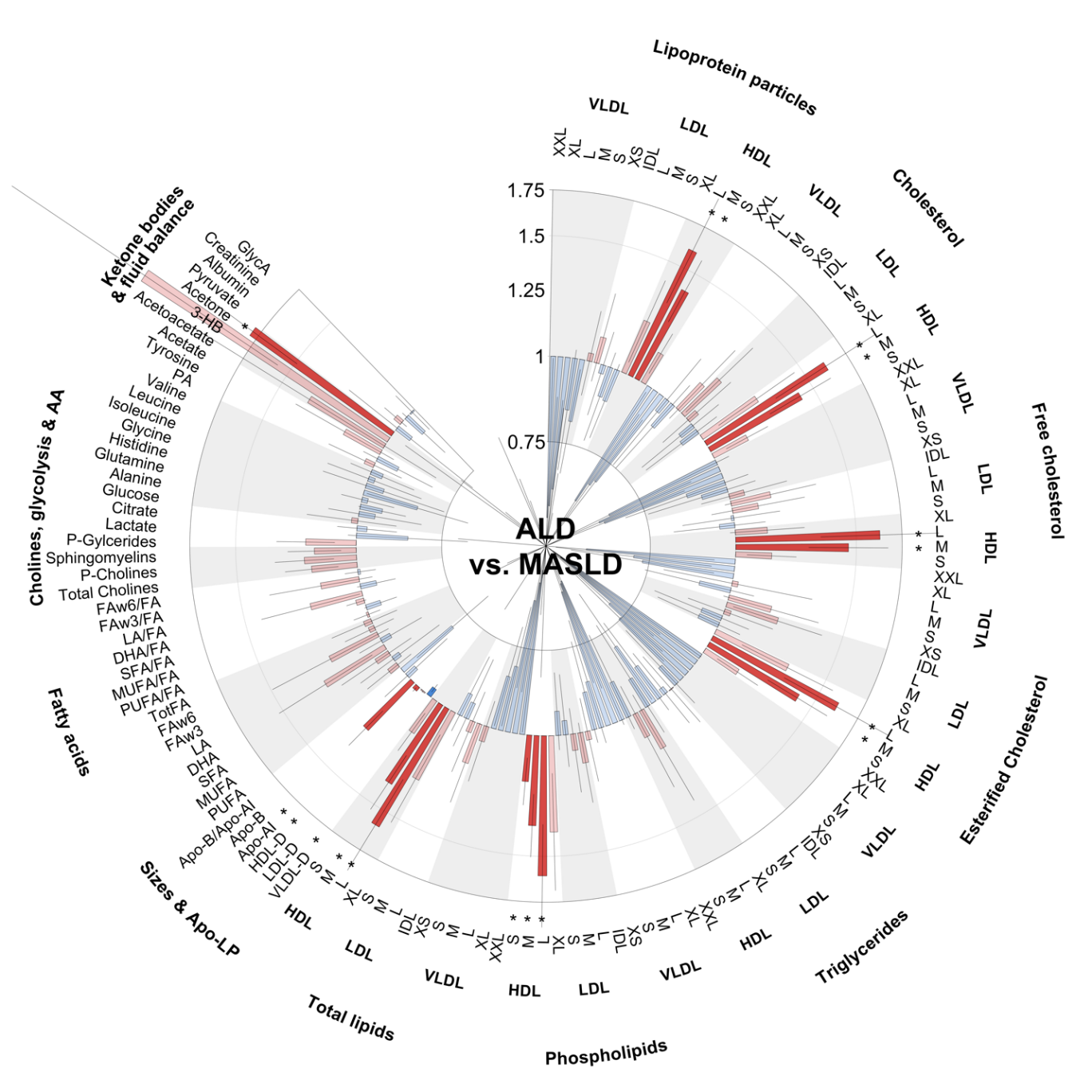
**

**Supplemental Figure 8:** **Circle plot for lipidomic analysis for ALD vs MASLD UKB participants at follow up.** Red=Higher in ALD. blue = higher in MASLD. Lipidomic parameters were measured through NMR spectroscopy. Hazard ratios (with 95% confidence intervals) are presented per 1-SD higher metabolic biomarker on the natural log scale. stratified by age. sex. body mass index. and Townsend deprivation index. * The Bonferroni method was employed to correct for multiple comparisons (p<0.05/249). Original code by Diego J Aguilar-Ramirez. Abbreviations: Apo-A1 - Apolipoprotein A1. Apo-B - Apolipoprotein B. FA - Fatty Acids. FAw3 - Omega-3 Fatty Acids. FAw6 - Omega-6 Fatty Acids. HDL - High-Density Lipoprotein. IDL - Intermediate-Density Lipoprotein. LA - Linoleic Acid. LDL - Low-Density Lipoprotein. M - Medium (lipid particle size). MUFA - Monounsaturated Fatty Acids. PUFA - Polyunsaturated Fatty Acids. S - Small (lipid particle size). SFA - Saturated Fatty Acids. TG - Triglycerides. VLDL - Very Low-Density Lipoprotein. XL - Extra Large (lipid particle size). XXL - Extra Extra Large (lipid particle size). 3-HB - 3-Hydroxybutyrate. P-Glycerides - Phosphoglycerides. P-Cholines - Phosphatidylcholines. DHA - Docosahexaenoic Acid.


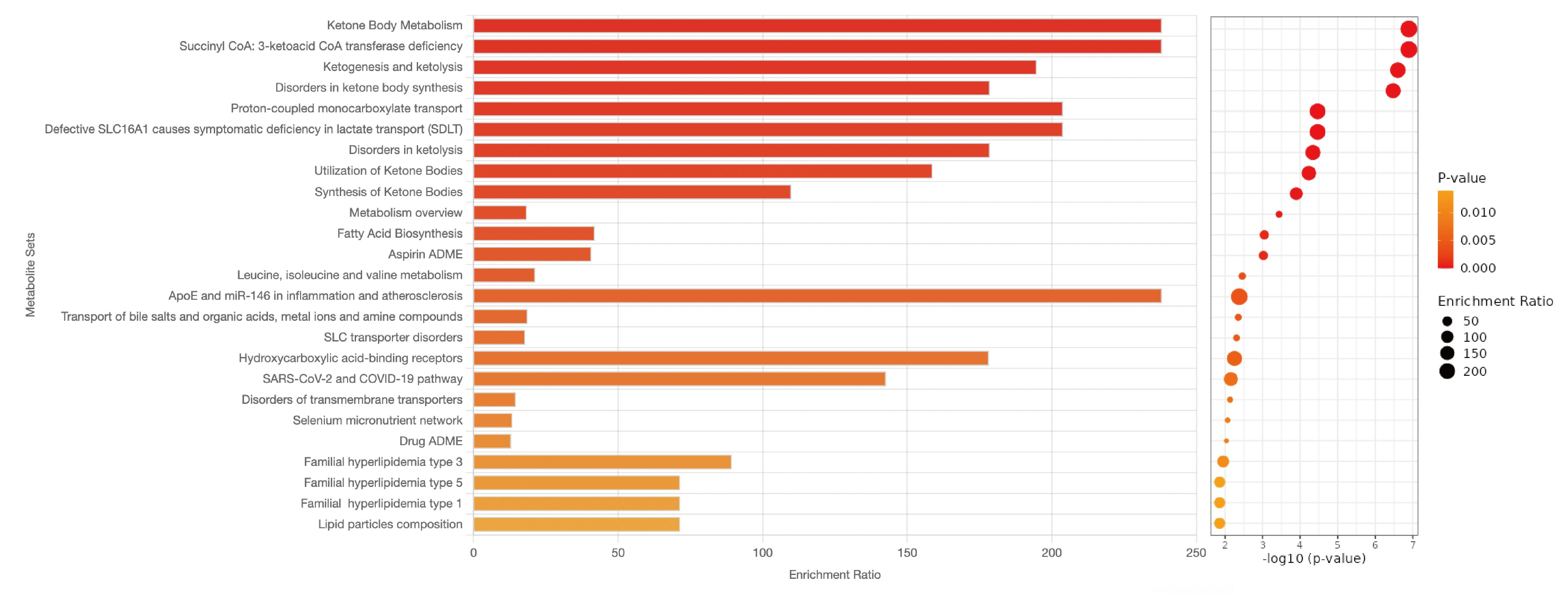


**Supplemental Figure 9:** **Enrichment analysis of metabolite and lipid pathways in MetaboAnalyst for MetALD cases in the UK Biobank.** Horizontal bar plot showing the top enriched pathways ranked by -log10(p-value). Bubble plot representing pathway enrichment results, with the size of the bubbles indicating the number of metabolites involved in each pathway and the color gradient reflecting the significance (-log10(p-value)) of enrichment


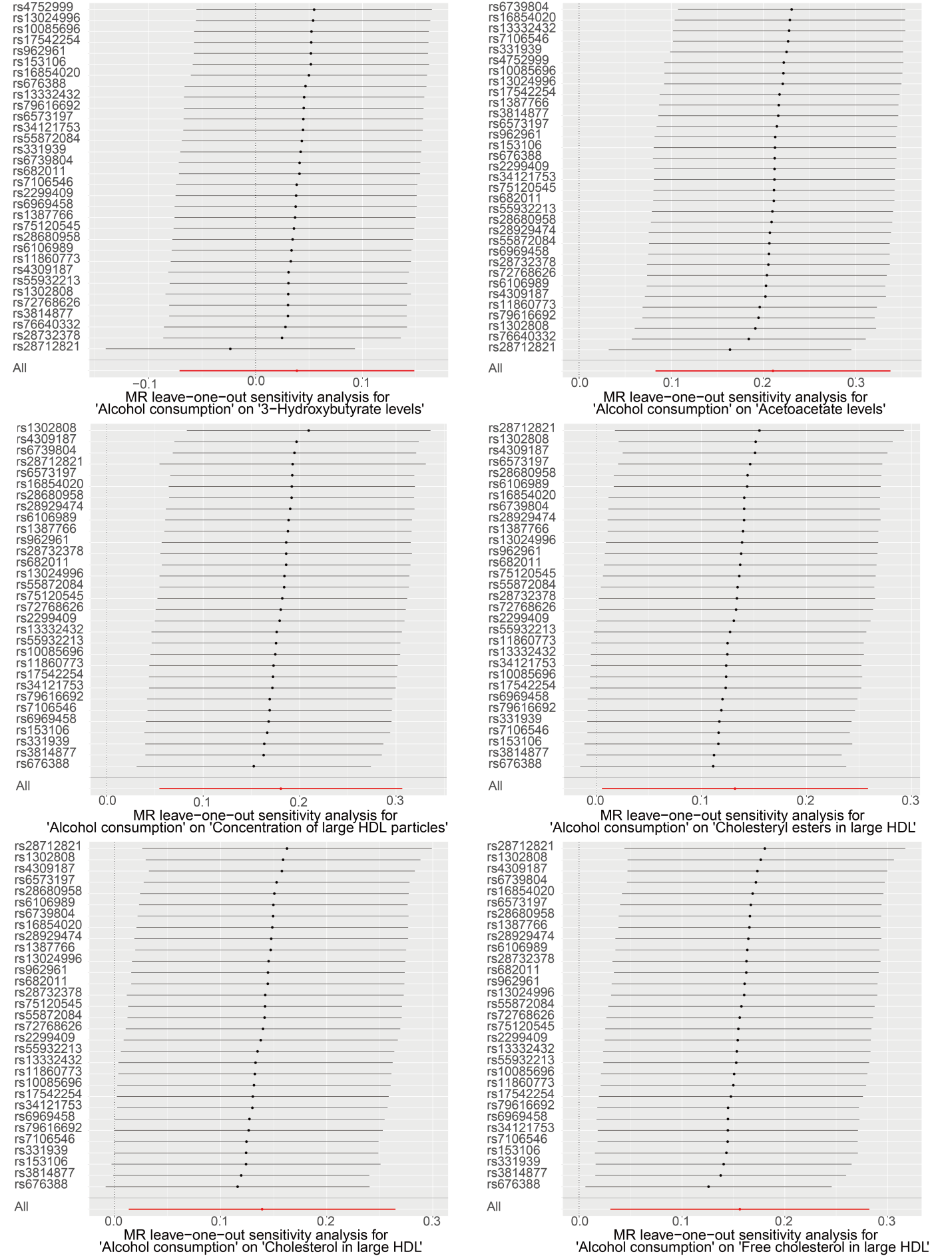


**Supplemental figure 10A: Leave-One-Out Sensitivity Analysis of Mendelian Randomization Results between alcohol consumption and top 30 discriminatory metabolites.**


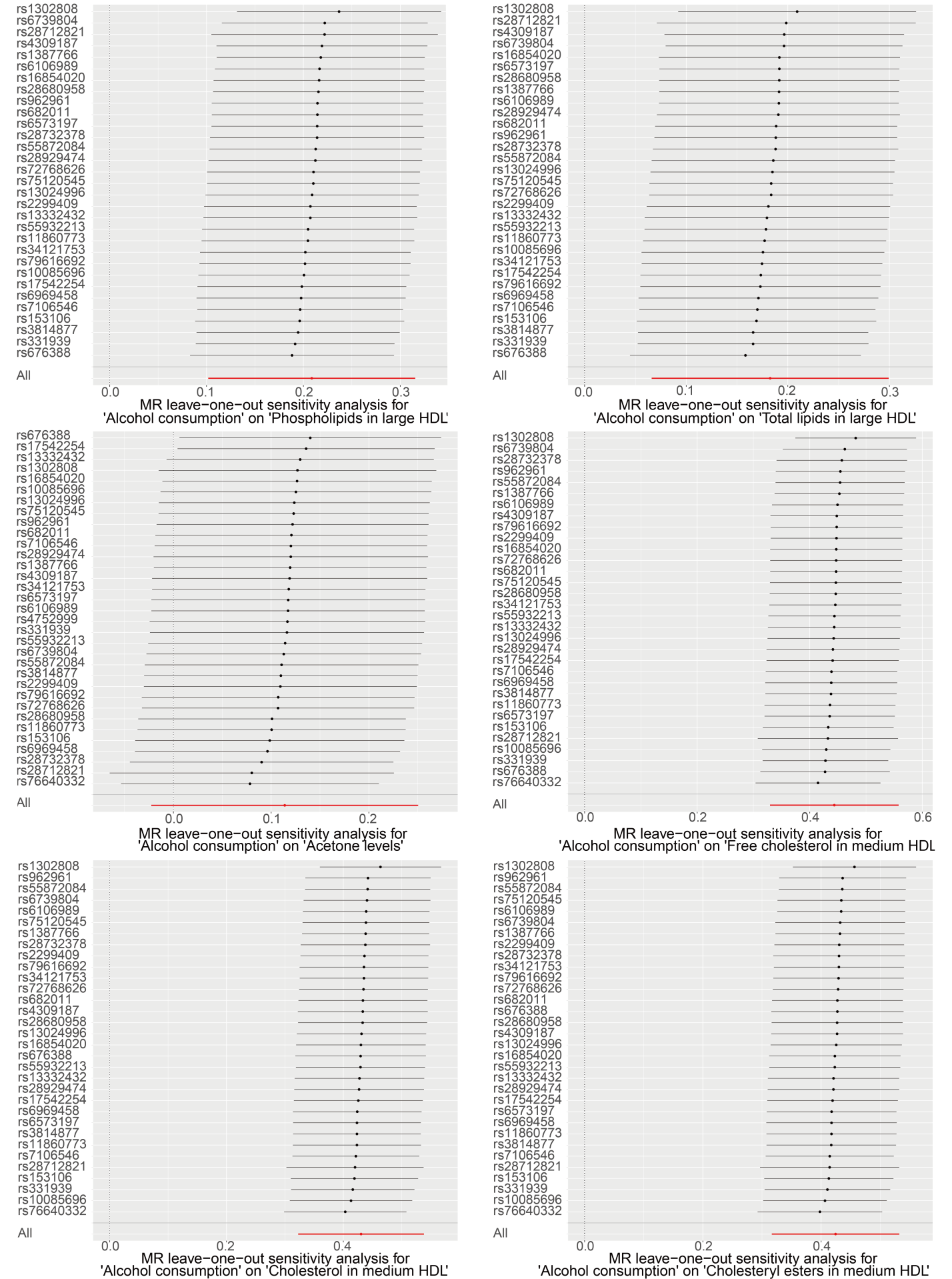


**Supplemental figure 10B: Leave-One-Out Sensitivity Analysis of Mendelian Randomization Results between alcohol consumption and top 30 discriminatory metabolites.**

**
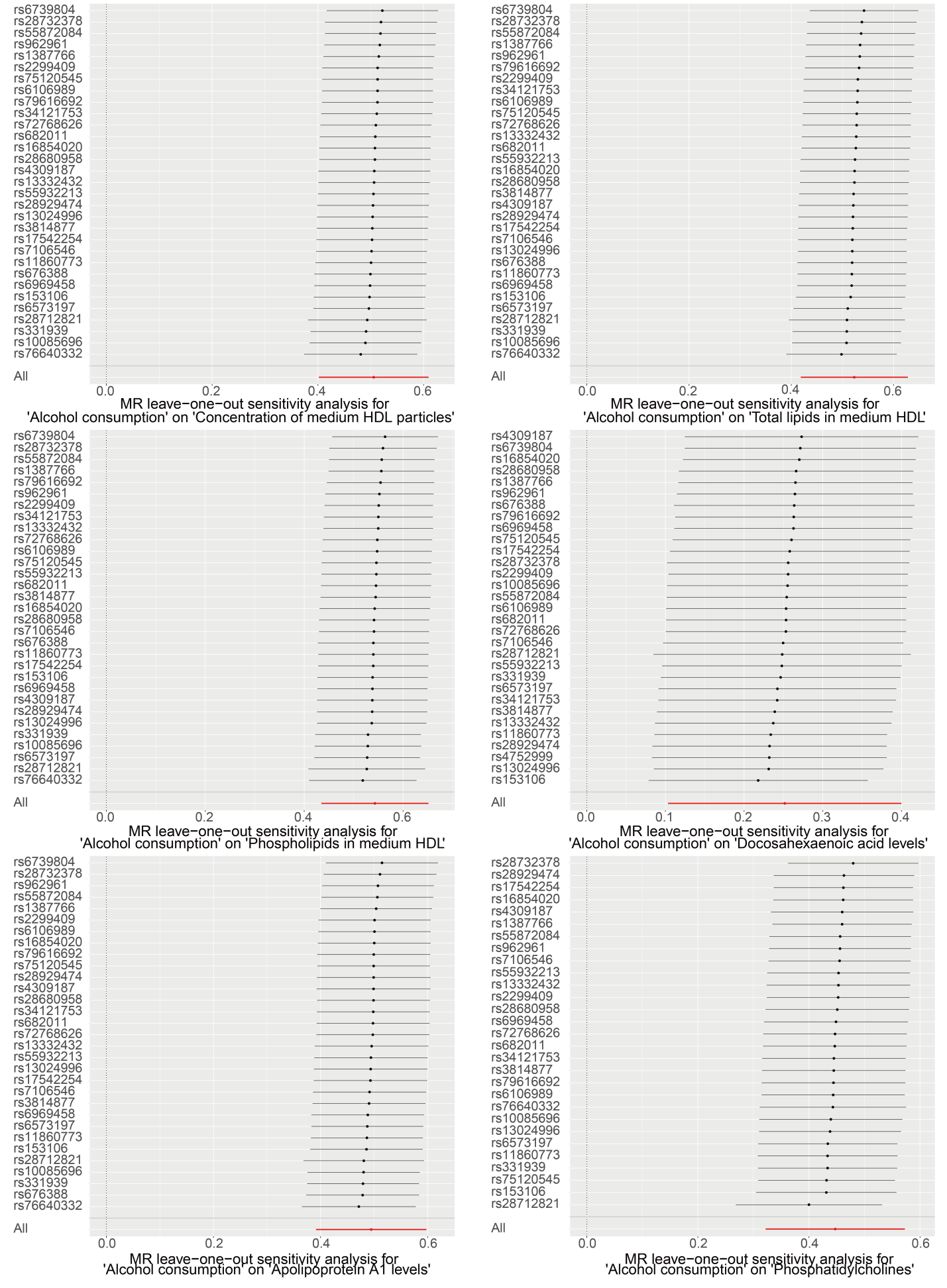
**

**Supplemental figure 10C: Leave-One-Out Sensitivity Analysis of Mendelian Randomization Results between alcohol consumption and top 30 discriminatory metabolites.**


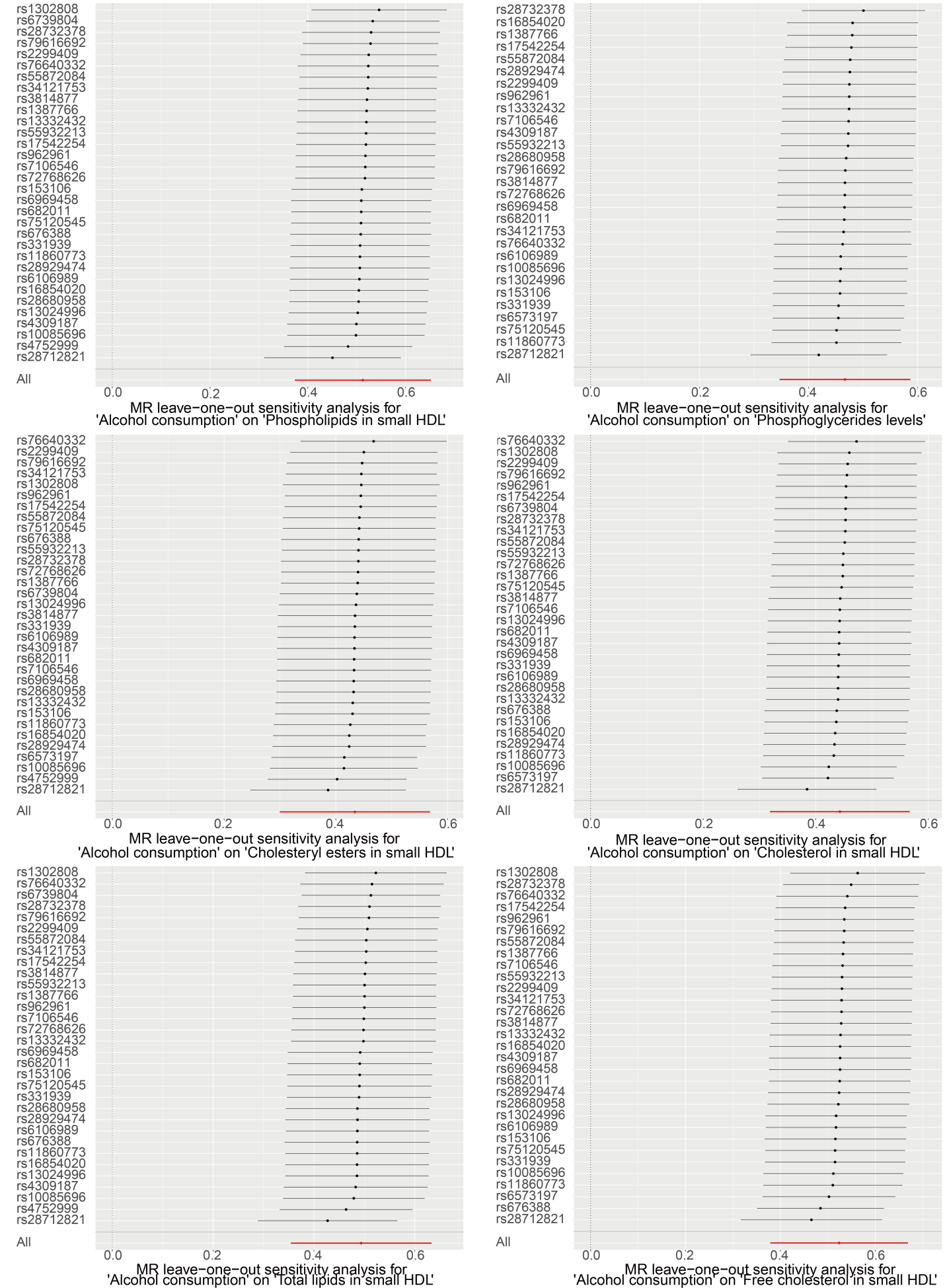


**Supplemental figure 10D: Leave-One-Out Sensitivity Analysis of Mendelian Randomization Results between alcohol consumption and top 30 discriminatory metabolites.**

**
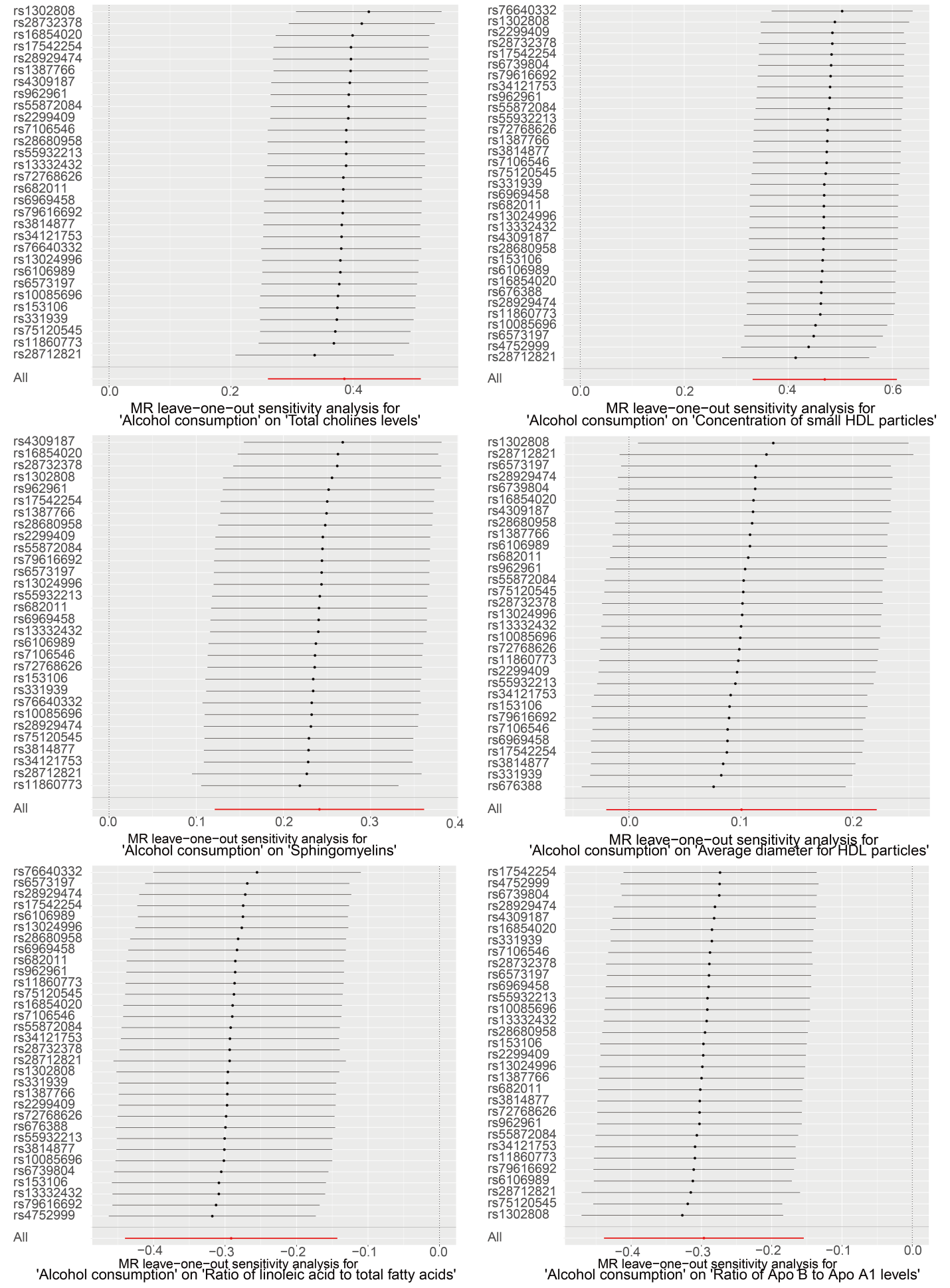
**

**Supplemental figure 10E: Leave-One-Out Sensitivity Analysis of Mendelian Randomization Results between alcohol consumption and top 30 discriminatory metabolites.**
